# CD47 Promotes, While Exposure to Apoptotic Cells Destroys the Fusion Program of Differentiating Myoblasts

**DOI:** 10.1096/fj.202503809R

**Published:** 2026-02-12

**Authors:** Maysaa Adil Ali, Albert Bálint Papp, Éva Garabuczi, Áron Károly Gere, Beatrix Dienes, Mónika Gönczi, György Vámosi, Krisztina Köröskényi, László Csernoch, Zsuzsa Szondy, Zsolt Sarang

**Affiliations:** ^1^ Faculty of Medicine, Doctoral School of Molecular Cellular and Immune Biology University of Debrecen Debrecen Hungary; ^2^ Department of Biochemistry and Molecular Biology, Faculty of Medicine University of Debrecen Debrecen Hungary; ^3^ Department of Biotechnology, College of Applied Sciences University of Technology Baghdad Iraq; ^4^ Faculty of Dentistry, Doctoral School of Dental Sciences University of Debrecen Debrecen Hungary; ^5^ Department of Integrative Health Science, Faculty of Health Science, Institute of Health Science University of Debrecen Debrecen Hungary; ^6^ Faculty of Medicine, Doctoral School of Molecular Medicine University of Debrecen Debrecen Hungary; ^7^ Department of Physiology, Faculty of Medicine University of Debrecen Debrecen Hungary; ^8^ Department of Biophysics and Cell Biology, Faculty of Medicine University of Debrecen Debrecen Hungary; ^9^ Division of Dental Biochemistry, Department of Basic Medical Sciences, Faculty of Dentistry University of Debrecen Debrecen Hungary

**Keywords:** apoptotic cells, CD47, “don't‐eat‐me” signals, efferocytosis, myoblast differentiation, myoblast fusion, phosphatidylserine, PIEZO1, Sirp1α, thrombospondin‐1

## Abstract

Cell fusion requires the activity of several phagocytic receptors and the temporary exposure of phosphatidylserine (PS) on the surface of viable myoblasts. Recently, we reported that these receptors turn myoblasts into potent phagocytic cells. Since cell fusion and phagocytosis share many molecules and mechanisms in myoblasts, we aimed to investigate how myoblasts choose between the two pathways during fusion. To prevent accidental uptake, viable cells express “don't eat‐me” signals. By analyzing RNA sequencing data, we found that differentiation affected the expression of multiple “don't eat‐me” genes in the C2C12 mouse myoblast cells, including upregulation of Sirpα, a receptor for CD47. The same was observed in differentiating myoblasts in vivo following cardiotoxin‐induced injury in mouse skeletal muscle. Treatment of differentiating C2C12 cells with anti‐CD47 antibody significantly reduced cell fusion but did not affect cell survival or differentiation. Both CD47 and SIRPα appeared at contact points of fusing myoblasts. Blocking CD47 signaling increased the uptake of viable red blood cells but only slightly increased the uptake of viable myoblasts. Blocking thrombospondin‐1, another CD47 ligand, also inhibited fusion. Inhibiting CD47 signaling did not impact the engulfment of apoptotic cells. However, long‐term exposure to continuously PS‐expressing apoptotic cells disrupted myotube formation by inhibiting PIEZO1 activation, leading to syncytia formation. Overall, our data show that differentiating myoblasts upregulate CD47 to avoid accidental phagocytosis of live cells but mainly to promote myoblast fusion. Therefore, the activity of this signaling pathway contributes to the decision‐making between the two processes that would compete with each other during myoblast differentiation.

## Graphical abstract

(A) CD47, acting in a cis‐manner with integrins, promotes myoblast fusion. TSP‐1 serves as a bridging molecule for these proteins to recognize PS on the surface of the other fusing myoblasts. (B) Blocking of these signals by neutralizing antibodies reduces cell fusion. Inhibition of PIEZO1 activity by constantly expressed PS on apoptotic cells leads to abnormal fusion and syncytium formation.

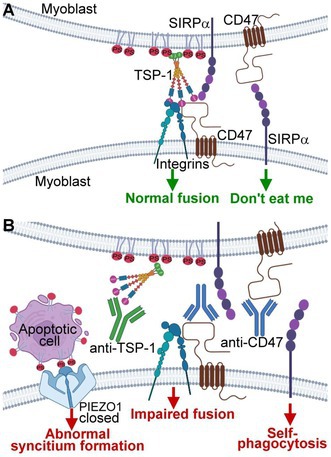

## 1 Introduction

The development and regeneration of skeletal muscle heavily depend on the differentiation of myoblasts guided by various myogenic regulatory factors (MRFs), such as myogenic factor 5 (Myf5), muscle‐specific regulatory factor 4 (Mrf4), myoblast determination protein (MyoD), and myogenin [1]. The differentiation process includes leaving the cell cycle, expression of muscle‐specific proteins, myoblast fusion, and elongation into long, cylindrical, multinucleated myofibers [2]. Even though transcriptional control is the key mechanism in myoblast differentiation, the steps of fusion and elongation of myoblasts to form myofibers are regulated by a multitude of membrane and cytosolic proteins [3]. The fusion itself starts with the alignment of myoblast (and myotube) membranes, followed by rearrangements of the actin cytoskeleton at the contact sites, and then by the membrane fusion [3]. The process depends on temporary phosphatidylserine (PS) exposure on the fusing cell surface [4, 5, 6]. Besides expressing the muscle‐specific fusion proteins, myomaker and myomerger [7], for binding PS and promoting membrane alignment, myoblasts display several receptors and bridging molecules traditionally expressed by macrophages, dendritic cells, and other professional phagocytes [8]. As soon as the membranes are aligned and fusion is initiated, PS is removed from the myoblast cell surface by the phospholipid flippase complex of ATP11A and CDC50A, inhibition of PIEZO1 by PS is terminated, and PIEZO1 initiates actomyosin fiber assembly beneath the plasma membrane to prevent uncontrolled fusion which would lead to abnormally shaped syncytia formation, and to generate a lateral compression force to support polarized elongation characteristic of myotubes [9]. Apoptotic cells also express PS on their surface as “eat‐me” signals [10], and these PS receptors on phagocytes mediate their engulfment (efferocytosis) [11]. Though in myoblasts, they were reported to participate primarily in cell adhesion, fusion, and survival [3, 8, 12, 13, 14, 15], recently, we have demonstrated that C2C12 myoblasts are also capable of apoptotic cell phagocytosis [16]. Since myoblasts express several phagocytic receptors and are capable of dead cell uptake, furthermore, fusion‐capable myoblasts display PS on their surface, the question arises, how these cells decide between engulfment and myoblast fusion during myotube formation.

To prevent accidental phagocytosis, mouse macrophages carry receptors to recognize “don't eat‐me” signals expressed by viable cells but lost by apoptotic cells. These receptors include SIRPα to detect CD47 [17], sialic‐acid‐binding immunoglobulin‐like lectin (SIGLEC)‐G to detect CD24 [18], paired immunoglobulin‐like receptor B (PIR‐B) to detect MHC class I/β2‐microglobulin molecules (B2m) [19], CD300a to detect PS [20], or the cell adhesion molecule CD31, which is activated via homophilic interactions [21]. These macrophage receptors are all ITIM‐containing receptors, which, following tyrosine phosphorylation, recruit SHIP1/2 tyrosine phosphatases to inhibit the efferocytosis signaling pathway, except CD31, for which a different mechanism of action has been proposed [22]. Previous studies have shown that disabling the “don't eat‐me” signaling pathways by administration of receptor‐ or ligand‐targeting antibodies strongly enhances both viable cell uptake [17] and tumor cell engulfment by the tumor‐infiltrating macrophages [23]. The present study aimed to determine whether, during myotube formation, “don't eat‐me” signaling is involved in choosing between initiating efferocytosis or cell fusion by fusion‐capable myoblasts.

## 2 Methods

All reagents were obtained from Merck (Darmstadt, Germany) except when indicated otherwise.

### 2.1 C2C12 Cell RNA Sequencing Data Processing

Gene expression data, generated from GPL24247 Illumina NovaSeq 6000 ( *Mus musculus* ), corresponding to five undifferentiated and five 6‐day differentiated C2C12 cell (RRID:CVCL_0188) samples, was retrieved from the public Gene Expression Omnibus (GEO, RRID:SCR_005012) repository (GEO series GSE215627). For a detailed description of the sample preparation and sequencing protocol, see the original paper by Lyu and Jiang [24]. Genes with FPKM (Fragments per kilobase of transcript per million mapped fragments) values equal to or less than 0.3 in every sample were considered negligibly expressed and absent. FPKM values were converted to TPM (transcript per million) by dividing each FPKM value by the sum of all FPKM values of the respective sample, and multiplying this by 10^6^. The gene expression list was imported into Microsoft Access and cross‐referenced to a list of known don't eat‐me signal‐related genes in mice (based on a literature search). *Z*‐score was calculated for every gene and plotted using the http://www.heatmapper.ca/expression/ web tool.

### 2.2 C2C12 Cell Culture and Differentiation

Murine myoblast C2C12 cells were obtained from American Type Culture Collection (ATCC; CRL‐1772), and were maintained according to the company's instructions. In brief, cells were cultured in Dulbecco's modified Eagle's medium (DMEM) supplemented with 20% fetal bovine serum (FBS), 100 U/mL penicillin, and 100 μg/mL streptomycin (growth medium) at 37°C in 5% CO_2_ and 95% air at 100% humidity. The absence of mycoplasma was tested using PCR Mycoplasma Test Kit I/C (PromoCell, Heidelberg, Germany, RRID:SCR_023579). Cells were plated into 24‐, 48‐, or 96‐well plates at a density of 3500 cells/cm^2^. For the 6‐day differentiation period, the cells were plated on a collagen‐coated surface and cultured in DMEM medium containing 2% FBS and 1% ITS (insulin, transferrin, sodium selenite), replaced every 2nd day with a fresh one. In some experiments, C2C12 cells were fed with apoptotic thymocytes, with RBCs, with 1 day differentiated myoblasts, with carboxylated latex beads (L3030) or treated with 1:100 dilutions (10 μg/mL) of neutralizing anti‐CD47 (Thermo Fisher Scientific, Waltham, MA, USA, 16–0471‐85, RRID:AB_2016575), neutralizing anti‐TSP‐1 (BA24, clone A6.1) or anti‐MHC‐I (16‐5957‐82, Thermo Fisher Scientific, RRID:AB_10670098) antibodies for various time periods before the subsequent experiments.

### 2.3 Cardiotoxin‐Induced Muscle Injury Model

For the muscle injury experiments, adult male 3‐month‐old C57BL/6 mice (Charles River Laboratories, Germany, RRID:IMSR_JAX:000664) were used, which were maintained in a 12 h light/12 h darkness cycle under SPF conditions, and had access to food and water ad libitum. For the selection of the animals, no inclusion or exclusion criteria were made. Animals from each cage were randomly allocated to the control or treated groups, but no blinding was used. Before inducing muscle injury, mice were anesthetized with 2.5% isoflurane using a SomnoSuite (Kent Scientific, Torrington, Connecticut, USA) device. The muscle damage was induced by injecting into the tibialis anterior (TA) muscle 50 μL of 12 μM CTX (Latoxan, Valence, France), dissolved in phosphate‐buffered saline. This concentration of CTX induces severe muscle injury, facilitating detection of more significant alterations in the subsequent regeneration process in the absence of regeneration–related genes, but still allows full regeneration as detected 3 months after the injury. All enrolled mice finished the study, that is, there was no attrition. Mice were sacrificed with isoflurane overdose at various time points following injury, and TA muscles were harvested and processed for further experiments. Sample size was based on estimations by power analysis with a level of significance of 0.05 and a power of 0.8 and was the same as reported by others in similar experiments [25]. Study protocols were approved by the Animal Care and Use Committee of the University of Debrecen, under permission number 7/2021/DEMÁB. All methods were carried out in accordance with relevant guidelines and regulations, and are reported in accordance with the ARRIVE guidelines.

### 2.4 Isolation of Muscle‐Derived CD45 ^+^ Leukocytes

Muscle‐derived CD45^+^ cell isolation was carried out as described previously [14]. Briefly, TA muscles were removed from control mice and at 2, 3, 4, 10, and 22 days post‐injury and dissociated in RPMI containing 0.2% collagenase II (Thermo Fisher Scientific) at 37°C for 1 h and filtered stepwise through 100 and 40 μm filters. CD45^+^ cells were isolated using magnetic sorting (Miltenyi Biotec, Gladbach, Germany).

### 2.5 Gene Expression Analysis

Total RNA from C2C12 cells or from the injured muscles was isolated with TRIzol (Thermo Fisher Scientific) reagent according to the manufacturer's instructions. Total RNA was reverse transcribed into cDNA using a High‐Capacity cDNA Reverse Transcription Kit (Thermo Fisher Scientific) according to the manufacturer's instructions. Quantitative reverse transcription polymerase chain reaction (RT–qPCR) was carried out in triplicate using pre‐designed TaqMan assays (Thermo Fisher Scientific) on a Roche LightCycler LC 480 real‐time PCR instrument. Relative mRNA levels were calculated using the comparative CT method and were normalized to Casein Kinase 2 Alpha 2 (Csnk2a2) and cytidine monophospho‐N‐acetylneuraminic acid synthetase (Cmas) mRNAs in the case of C2C12 cells and TA muscles, respectively. Catalog numbers of the TaqMan assays used were the following: Csnk2a2 Mm00441242_m1, Myomaker Mm00481256_m1, MyoD1 Mm00440387_m1, Myog Mm00446194_m1, Myh1 Mm01332489_m1, Cd47 Mm00495011_m1, Cmas Mm00515534_m1, Pax7 Mm00834082_m1, Thbs1 Mm00449032_g1, and Sirpa Mm00455928_m1.

### 2.6 Immunofluorescent Staining of Muscles

TA muscles from control mice or TA muscles at the indicated days post‐injury were dissected for histological assessment. The muscles were snap‐frozen in liquid nitrogen‐cooled isopentane and kept at −80°C. Seven‐micrometer‐thick cryosections were cut at −20°C using a 2800 Frigocut microtome (Leica, St Jouarre, France) and were kept at −20°C until further analysis. To detect CD47 or SIRPα, frozen muscle sections were incubated in 10 mM citric acid‐sodium citrate buffer (pH 6.0) for 15 min, then in blocking solution (50% FBS in PBS) for 1 h at room temperature. After blocking, samples were labeled with Dylight 488 conjugated anti‐laminin B (PA5‐22901, Invitrogen, Carlsbad, CA, USA) (1:100) and anti‐CD47 or anti‐SIRPα (Thermo Fisher Scientific, 16‐1721‐85, RRID:AB_10852704) antibodies (1:100) at 4°C overnight, followed by addition of Alexa‐546‐conjugated anti‐rat secondary antibodies (6 μg/mL) (Thermo Fisher Scientific, A‐11081, RRID:AB_2534125). The slides were then washed three times with PBS and were mounted with glass coverslips. Images were taken on a FLoid Cell Imaging Station fluorescent microscope (Thermo Fisher Scientific) and analyzed using ImageJ v1.52 software (RRID:SCR_003070, National Institutes of Health, Bethesda, MD, USA) with a muscle morphometry plugin. Areas containing fibers with centrally located nuclei were considered regenerating muscle parts.

### 2.7 Cell‐Surface Detection of CD47, SIRPα, and PIEZO1

Proliferating and 6‐day differentiated C2C12 cells were detached by enzyme‐free Cell dissociation buffer (Thermo Fisher Scientific), centrifuged, and incubated in 50% FBS/PBS containing 10 μg/mL non‐specific IgG (sc‐3888, Santa Cruz Biotechnology, Dallas, Texas, USA) for 20 min on ice. After centrifugation, cells were labeled with 10 μg/mL rat anti‐CD47 (16‐0471‐85) or rat anti‐SIRPα (16‐1721‐85) or rat isotype control (Thermo Fisher Scientific, 10700) antibodies dissolved in 10% FBS/PBS (FACS buffer) for 20 min on ice. Following washing with FACS buffer, cells were labeled with Alexa Fluor 546‐conjugated anti‐rat antibodies (6 μg/mL) (A‐11081, RRID:AB_2534125) for 20 min on ice. For cell surface PIEZO1 detection, cells were labeled with anti‐Piezo1 extracellular domain (4 μg/mL) (15939‐1‐AP, Proteintech, Rosemont, IL, USA) or rabbit isotype control (sc‐3888) antibodies dissolved in FACS buffer for 60 min on ice. Following washing with FACS buffer, cells were labeled with Alexa Fluor 488‐conjugated anti‐rabbit IgG (10 μg/mL) (Thermo Fisher Scientific) for 60 min on ice. After labeling, cells were washed with FACS buffer, and fluorescence was measured on Becton Dickinson FACSCalibur (RRID:SCR_000401, Becton Dickinson Company, Franklin Lakes, NJ, USA). Apoptotic thymocytes and C2C12 cells were gated according to their size. FACS analyses demonstrating that apoptotic thymocytes expressed such a low level of PIEZO1 that it did not affect the detection of PIEZO1 expression of myoblasts, and how high and low PIEZO1‐expressing myoblasts were gated are shown in Figure S1.

### 2.8 Western Blot Analysis of MYH4 Expression in Differentiating C2C12 Cells

C2C12 cells were collected and lysed in ice‐cold lysis buffer (50 mM Tris, 1 mM EDTA, MEA, 0.5% Triton X‐100, 1 mM PMSF) containing a protease inhibitor cocktail (Sigma‐Aldrich) at a 1:100 dilution, homogenized with 5 strokes of a sonicator at 40% cycle intensity (Branson Sonifer 450), after that centrifuged at 15 000 rpm, at 4°C for 15 min. Protein concentrations were measured in 96‐well plates with the Bradford assay (Bio‐Rad, Budapest, Hungary). Samples were mixed with loading buffer and incubated at 100°C for 10 min. 20 μg protein from each sample was separated on 8% SDS‐polyacrylamide gels, blotted onto a PVDF membrane (Millipore), blocked with 5% non‐fat dry milk in 1× Tris‐buffered saline containing Tween 20 (TTBS) for 1 h at room temperature, and incubated overnight at 4°C with primary antibodies diluted in 0.5% milk in TTBS: monoclonal anti‐MYH4 antibody (53‐6503‐82, Thermo Fisher Scientific, RRID:AB_10671272) in 1:2000, monoclonal anti‐GAPDH antibody (MA5‐15738, Thermo Fisher Scientific, RRID:AB_10977387) in 1:5000 dilution. After washing four times with 1× TTBS, membranes were incubated for 1 h with Anti‐Mouse IgG (H + L) Antibody [HRP] (lab0252, Covalab, Cambridge, UK) in a 1:10 000 dilution. After washing, the protein bands were visualized with an ECL kit (Advansta) and quantified using ImageJ software version 2.0.0.

### 2.9 Annexin V Staining

1‐ or 2‐day differentiated C2C12 myoblasts were detached by enzyme‐free Cell dissociation buffer, centrifuged, and resuspended in 50 μL PBS containing 2 mM calcium chloride (binding buffer) and incubated with 2 μL annexin V‐Alexa Fluor 647 (Thermo Fisher Scientific) for 15 min at room temperature. After this, 350 μL binding buffer was added to the cells, and fluorescence was measured on Becton Dickinson FACSCalibur.

### 2.10 Cell Viability Determination

To determine the cell numbers, C2C12 cells were seeded onto 96‐well plates in growth medium and treated with anti‐CD47 or anti‐MHC‐I antibodies for 24 to 72 h. Non‐toxic, cell‐permeable PrestoBlue (Thermo Fisher Scientific) dye was added to the wells (1:10), and absorbance was measured at the indicated time points on a Synergy H1 microplate reader (Thermo Fisher Scientific) at 570/60 nm (excitation/emission). Dead cells with permeable membranes were stained with 10 μg/mL propidium iodide for 5 min, and photos were taken on a FLoid Cell Imaging Station (Thermo Fisher Scientific), and the number of propidium iodide‐positive cells/optical fields was counted for eight wells.

### 2.11 Generation of Bone Marrow–Derived Macrophages (BMDMs)

Bone marrow progenitors were obtained from the femur of C57BL/6 mice by lavage with sterile physiological saline. Cells were differentiated for 5 days in DMEM medium supplemented with 10% conditioned medium derived from L929 cells (RRID:CVCL_0462), as a source for macrophage colony‐stimulating factor, and 2 mM glutamine, 100 U/mL penicillin, and 100 μg/mL streptomycin at 37°C in 5% CO_2_. Non‐adherent cells were washed away every second day.

### 2.12 Generation of Apoptotic Thymocytes

Thymi from 4‐week‐old C57BL/6 mice were collected and placed in 10 mL physiological saline solution. Thymocytes were separated by gently pushing the thymus through a metal mesh filter. The cells were then filtered through a 41 μm filter and centrifuged for 8 min at 200 g. The pellet was resuspended in 10 mL RPMI 1640 media supplemented with 2 mM glutamine, 100 U/mL penicillin, and 100 μg/mL streptomycin in the absence of FBS and incubated for 20 h (10^7^ cells/mL). This treatment results in approximately 80% annexin‐positive cells [26]. Apoptotic thymocytes were then stained with 0.5 μM CellTracker Deep Red dye (Thermo Fisher Scientific) at 37°C for 30 min in the absence of FBS, washed, and added to C2C12 cell cultures or to BMDMs.

### 2.13 Red Blood Cell (RBC) Isolation

A drop of blood was collected from the tail vein of C57BL/6 mice and mixed with 200 μL physiological saline solution. Cells were centrifuged for 5 min at 200 *g*. The pellet was resuspended in 200 μL PBS and stained with pHrodo green dye (Thermo Fisher Scientific) according to the manufacturer's instructions.

### 2.14 In Vitro Efferocytosis Assay of Proliferating C2C12 Myoblasts and BMDMs

Proliferating C2C12 cells and BMDMs were grown in 48‐well plates as described above. pHrodo green‐stained viable RBCs or Deep Red‐stained apoptotic thymocytes were added to the C2C12 cells or to BMDMs in a 5:1 (target/phagocytosing cells) ratio on the day of the flow cytometric measurement for 60 min. After coculture, non‐engulfed RBCs or apoptotic thymocytes were washed away extensively with PBS, and C2C12 cells and BMDMs were detached by trypsinization at 37°C for 15 min and filtered through a 100 μm filter before the measurement to eliminate cell clots. The percentage of engulfing cells was determined on a Becton Dickinson FACSCalibur flow cytometer. The obtained data were analyzed using the web‐based program floreada.io (https://floreada.io).

### 2.15 Immunostaining for Determining Myoblast Fusion or Efferocytosis of Apoptotic Thymocytes by Fusing Myoblasts

For MYH4 staining, 6‐day differentiated C2C12 cells in 48‐well plates were fixed with ice‐cold methanol for 15 min at 4°C, washed three times with PBS, and then blocked with PBS/5% FBS/0.3% Triton X‐100 for 60 min at room temperature. Alexa Fluor 488 conjugated anti‐Myosin heavy chain 4 (Thermo Fisher Scientific) was added at a 1:100 dilution in PBS/1% bovine serum albumin/0.3% Triton X‐100 for 12 h at 4°C. After staining, cells were rinsed three times in PBS and counterstained with NucBlue according to the manufacturer's instructions for DNA staining. Fluorescent and bright‐field pictures were taken on a FLoid Cell Imaging Station fluorescent microscope. To evaluate myoblast fusion, digitally captured photos were analyzed using ImageJ software. The fusion index was calculated by expressing the number of nuclei within MYHC4‐positive myotubes with ≥ 3 nuclei as a percentage of the total nuclei. To evaluate efferocytosis of Deep Red‐stained apoptotic thymocytes were exposed to 5‐day differentiated C2C12 myoblasts, myoblasts were stained as above, and the number of cells taken up was determined by fields of view at the indicated times.

### 2.16 Confocal Microscopy

C2C12 myoblasts were seeded in collagen‐coated 8‐well Ibidi chamber slides (Idibi GmbH, Gräfelfing, Germany) and allowed to differentiate for 6 days. To detect viable myoblast uptake, on day 6, cells were coincubated with Deep Red‐stained 1‐day differentiated C2C12 myoblasts for 6 or 24 h in the presence or absence of anti‐CD47 antibody (10 μg/mL, RRID:AB_2016575). Following incubation, cells were immunostained for myosin heavy chain 4 and NucBlue. To assess cell fusion, 5‐day differentiated C2C12 cells in Ibidi chambers were coincubated with 3‐day differentiated, Deep Red‐stained myoblasts for 3 h. Following washing, the cells were immunostained for myosin heavy chain 4 and NucBlue. For CD47 and SIRP‐alpha colocalization, C2C12 cells were allowed to differentiate in Ibidi chambers for 3 days. Then they were fixed with ice‐cold methanol for 15 min at 4°C, washed three times with PBS, and then blocked with PBS/50% FBS and labeled with rat anti‐CD47 antibody (1:100) and rabbit anti‐SIRP‐alpha (1:100, Thermo Fisher Scientific, ma5‐29806, RRID:AB_2785628) in PBS at 4°C for 1 h. After this, cells were washed with PBS and incubated with Alexa‐546‐conjugated anti‐rat antibodies (6 μg/mL) and Alexa‐488‐conjugated goat anti‐rabbit IgG (6 μg/mL) (Thermo Fisher Scientific, A‐11034, RRID:AB_2576217) for 1 h. Following nuclear staining with NucBlue, images were taken with a Zeiss LSM 880 confocal microscope (Carl Zeiss, Jena, Germany) equipped with a 40×, NA 1.2 C‐Apochromat water immersion objective.

### 2.17 Immunostaining for Determining PIEZO1 Expression on Myoblasts

To perform immunocytochemistry, C2C12 cells (3 × 10^4^ cells/sample) were seeded into sterilized glass coverslips. Settled myoblasts were incubated with or without Deep Red‐stained apoptotic thymocytes (5 × 10^6^/sample) for 1 h at thermostated environment. After washing the cultures with PBS, they were fixed with 4% PFA for 15 min. 0.1 M glycine‐containing PBS (3 × 15 min) was used to neutralize excess formaldehyde, then cells were blocked with a serum‐free Protein blocking solution (DAKO) for 30 min. Primary polyclonal antibody against Piezo1 extracellular domain (15939‐1‐AP, Proteintech, RRID:AB_2231460) was diluted in blocking solution (1:250), added to the samples, and incubated overnight at 4°C in a humidity chamber. The next day, samples were washed three times with PBST and incubated with Alexa Fluor 488‐conjugated anti‐rabbit IgG (Thermo Fisher Scientific) (1:500) for 1 h at room temperature. After washing three times, a drop of mounting medium containing NucBlue was added to each slide. Confocal images were acquired with an AiryScan 880 laser scanning confocal microscope (Zeiss, Oberkochen, Germany) equipped with a 63× oil objective. Excitation at 488, 543, and 405 nm wavelengths was used to detect fluorescence of the aforementioned secondary antibody and fluorescent labeling, respectively, while emission was collected above 550 nm with a long pass filter.

### 2.18 Determination of the PIEZO1 Activity of Myoblasts in the Presence and Absence of Apoptotic Thymocytes

Cells were loaded with 10 mM Fura‐2‐AM (F1221, Thermo Fisher Scientific) for 30 min at 37°C, then kept in Tyrode's solution. PIEZO1 was activated by adding Yoda1 (25 μM) to the cells. CoolLED pE‐340fura light source (CoolLED LTD., Andover, England) mounted on a ZEISS Axiovert 200 M inverted microscope (Zeiss) was used to detect changes in fluorescence intensity. Myoblasts interacting with apoptotic thymocytes were selected prior to measurements to detect their [Ca^2+^]_I_ selectively. Fura‐2 was excited with wavelengths alternating between 340 and 380 nm, and the emission was detected with a band‐pass filter of 505–570 nm. The image acquisition and post‐processing were performed with the AxioVision (RRID:SCR_002677, rel. 4.8) software (Zeiss). The [Ca^2+^]_i_ was determined from the intensity ratio of images taken at 340 and 380 nm after background correction.

### 2.19 Statistical Analysis

All the data are representative of at least three independent experiments, and all data are expressed as individual values and mean or mean ± SD. Normal distribution of the data was tested using the online tool https://www.statscalculators.com/calculators/chart/qq‐plot. Statistical analysis was performed using two‐tailed, unpaired Student's *t*‐test for two‐group tests with normal data distribution and Mann–Whitney *U*‐test for non‐normal distribution samples, and one‐way ANOVA with post hoc Tukey HSD test for multiple comparisons. The equal variance of the samples was tested by *F*‐test. Statistical analysis was carried out using GraphPad Prism (v5, RRID:SCR_002798). *p < 0.05, **p < 0.01, ***p < 0.001, and ****p < 0.0001.

All the methods were performed according to the relevant guidelines and regulations.

## 3 Results

### 3.1 Myogenic Differentiation Alters the Expression of “Don't Eat‐Me” Signaling Molecules in Myoblasts

**FIGURE 1:**
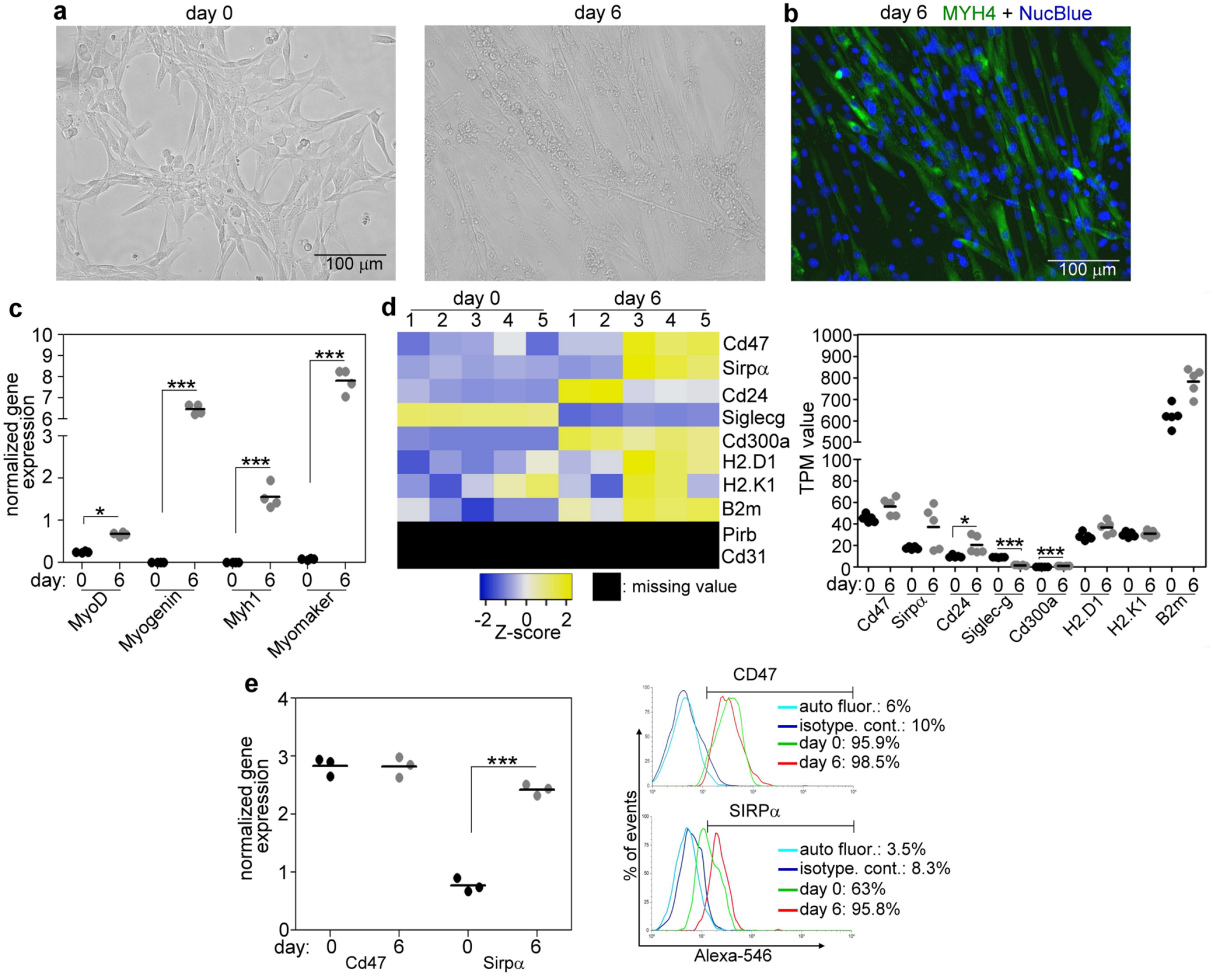
Myogenic differentiation alters the expression of phagocytosis‐associated genes in C2C12 myoblasts. (a) Representative light microscopic picture of proliferating and 6‐day differentiated C2C12 myoblasts. (b) Representative immunofluorescence images of myosin heavy chain 4 (MYH4) (green) and NucBlue (blue) stained 6‐day differentiated C2C12 cells forming multinucleated myotubes. (c) The expression level of four myogenic differentiation markers in proliferating and 6‐day differentiated C2C12 cells determined by qRT‐PCR. (d) The left panel shows the heatmap of “don't eat‐me” signal‐associated gene expressions in proliferating and 6‐day differentiated C2C12 cells determined by RNA sequencing. The right panel shows the average TPM values of the detected don't eat‐me signal genes. (e) Left panel: the mRNA expression level of Cd47 and Sirpα in proliferating and 6‐day differentiated C2C12 cells determined by qRT‐PCR. Right panel: representative flow cytometry histograms showing the cell surface level of CD47 and SIRPα in proliferating and 6‐day differentiated C2C12 cells. Data are expressed as mean and individual values. Asterisks denote statistically significant differences from day 0 (*p < 0.05, ***p < 0.001, Student's *t*‐test). *n* = at least 3 samples.

In our experiments, an in vitro model of myoblast differentiation, the differentiation of C2C12 myoblast cells [27] was studied. The shift of growth medium to low‐serum fusion medium induces the differentiation of the proliferating C2C12 myoblasts, resulting in formation of multinucleated, myosin‐expressing myotubes, as demonstrated by visualization of myotubes, myosin heavy chain 4 (MYH4) immunostaining, and detecting differentiation markers, such as MyoD, myogenin, Myh1, and myomaker by qRT‐PCR (Figure 1a–c). When searching for the expression of “don't eat‐me” signaling molecules in C2C12 myoblasts using the gene expression data corresponding to five undifferentiated and five 6‐day differentiated C2C12 cell samples retrieved from the public Gene Expression Omnibus repository (GSE215627), we found the expression of some of them (Figure 1d). Thus, the Cd47/Sirpα, the Cd24/Siglec‐G ligand/receptor pairs and Cd300a were expressed. In contrast, neither Cd31 nor Pir‐B expression could be detected in the proliferating or differentiating myoblasts. While the expression of most of the “don't eat‐me” signals increased, that of SIGLEC‐G decreased during myoblast differentiation. The constant expression of CD47 and the increasing expression of SIRPα during myoblast differentiation, the highest expressed “don't eat‐me” signal pair in C2C12 cells, were confirmed by us on both mRNA and protein levels (Figure 1e).

### 3.2 The Expression of Sirpα Is Also Enhanced During In Vivo Myoblast Differentiation

**FIGURE 2:**
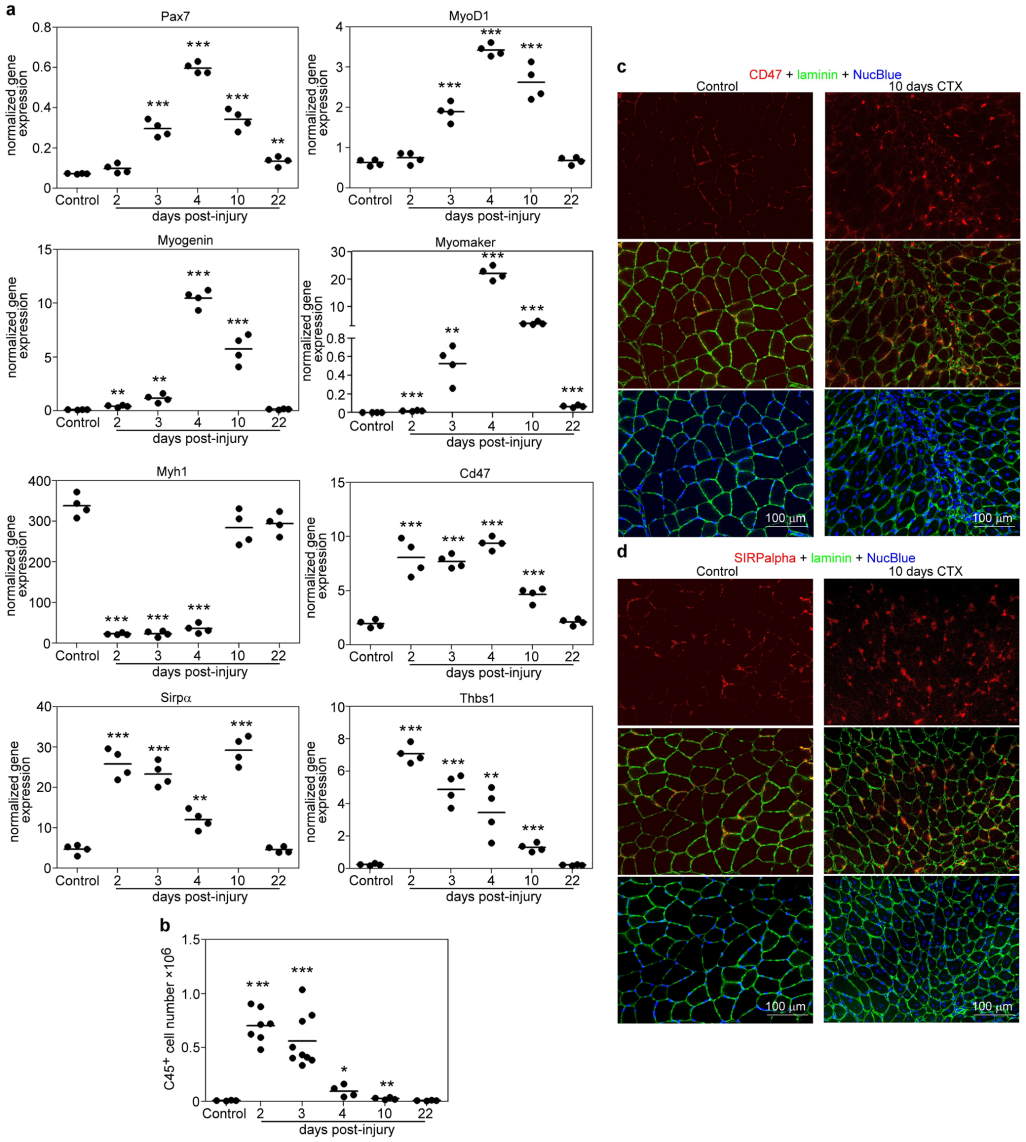
The expression of Cd47 and Sirpα increases during skeletal muscle regeneration. (a) mRNA expression levels of various myogenic genes in TA muscles determined by qRT‐PCR following CTX‐induced injury (*n* = 4). (b) Number of muscle‐infiltrating CD45^+^ cells in the regenerating TA muscles. Data are expressed as mean and individual values. Asterisks denote statistically significant differences from uninjured legs (*p < 0.05, **p < 0.01, ***p < 0.001, Student's *t*‐test). (c) Immunostaining for laminin B and CD47 in uninjured and 10‐day regenerating TA muscles. (d) Immunostaining for laminin B and SIRPα in uninjured and 10‐day regenerating TA muscles.

To demonstrate that the in vitro findings have in vivo relevance, we decided to follow the skeletal muscle expression of Cd47 and Sirpα in the cardiotoxin‐induced skeletal muscle injury model as well [28]. Within this model, following cardiotoxin injection of the tibialis anterior muscle of mice, a sterile inflammation is initiated with infiltrating inflammatory cells, which trigger the proliferation of Pax7‐expressing muscle stem cells (satellite cells). The early inflammatory response is followed by myoblast differentiation, fusion, and myofiber growth, which is completed by the third week. As seen in Figure 2a, cardiotoxin injury triggered the proliferation and differentiation of satellite cells, detected by the increased expression of Pax7, MyoD, myomaker, and myogenin. By day 10 post‐injury, the differentiation was completed as the expression of Myh1 levels reached the initial control levels, and the proliferation of stem cells, reflected in Pax7 expression, started to decrease. Previous studies from our laboratory have shown that around this day, a significant myoblast fusion activity can be detected [14, 15, 29]. CD47 and Sirpα are expressed by the infiltrating cells as well; thus, their expression during skeletal muscle regeneration showed an early peak reflecting the number of infiltrating inflammatory cells (Figure 2b). However, the highest Sirpα expression correlated with the myoblast fusion activities at day 10. To prove that these molecules are indeed expressed by myoblasts on the protein level as well, sections of the tibialis anterior muscle were stained for SIRPα and CD47 at day 0 and at day 10 following cardiotoxin injury. As demonstrated in Figure 2c,d, we could detect the increased expression of both proteins in the regenerating muscle as well. Altogether, the expression and modulation of the expression of these two “don't eat‐me” signal molecules imply a specific function of these proteins in the differentiating myoblasts.

### 3.3 Anti‐CD47 Antibody Treatment Impairs Myoblast Fusion in Differentiating C2C12 Cells

**FIGURE 3:**
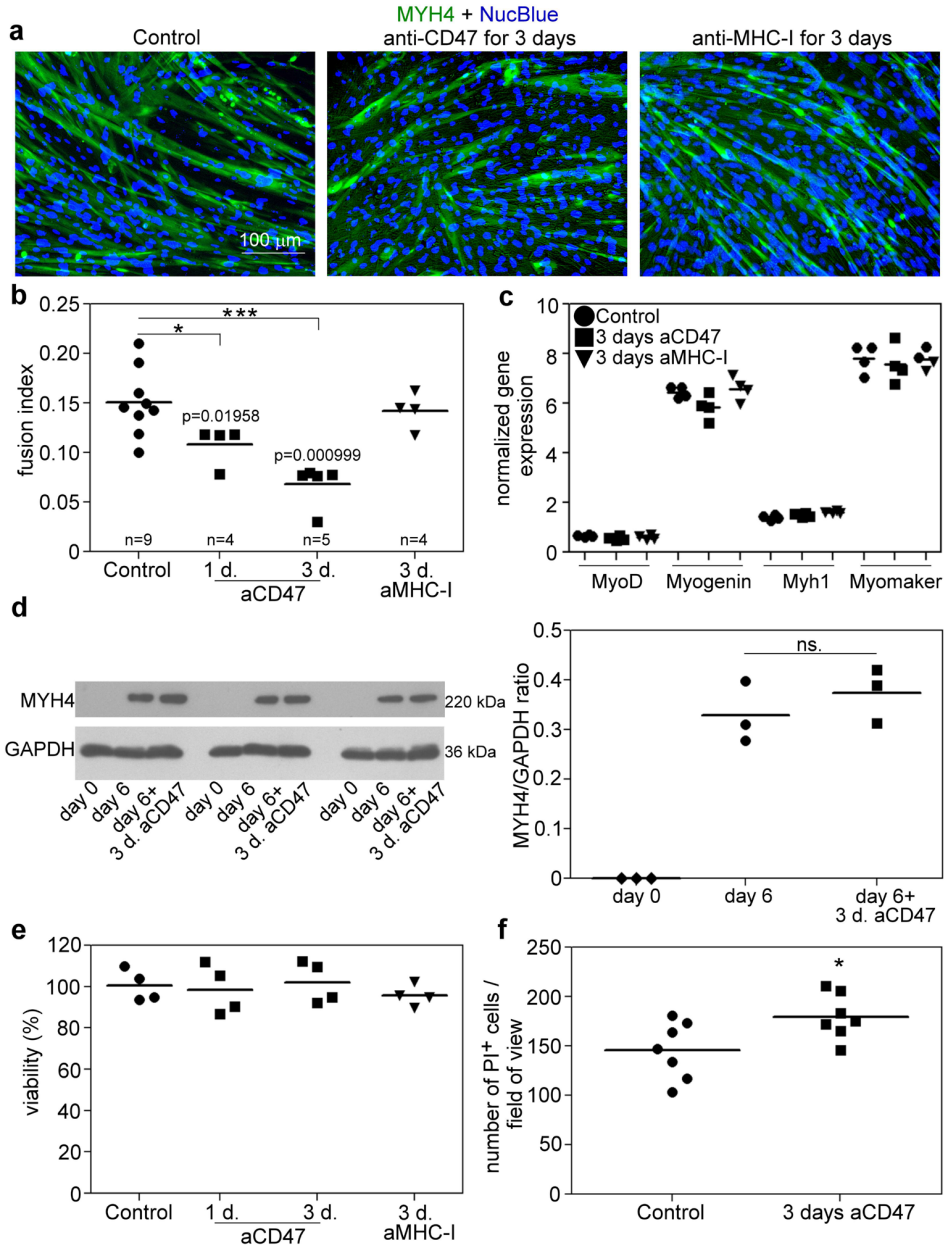
Anti‐CD47 antibody treatment impairs myoblast fusion in differentiating C2C12 cells. (a) Representative immunofluorescence images of myosin heavy chain 4 (MYH4) (green)‐ and NucBlue (blue)‐stained 6‐day differentiated C2C12 grown for 3 days in the absence or presence of anti‐CD47 or anti‐MHC‐I antibodies. (b) The effect of anti‐CD47 (added for the last 1 or 3 days) or anti‐MHC‐I (added for 3 days) antibodies on the fusion of 6‐day differentiated C2C12 cells. Asterisks denote statistically significant differences from the control (* *p* < 0.05, ***p < 0.001, Mann–Whitney *U*‐test). (c) The expression of myogenic differentiation markers in 6‐day differentiated C2C12 cells in the absence or presence of anti‐CD47 and anti‐MHC‐I antibodies (added for 3 days) determined by qRT‐PCR. (d) The expression of MYH4 in 6‐day differentiated C2C12 cells in the absence or presence of anti‐CD47 antibodies (added for 3 days) determined by Western blot analysis. (e) The effect of anti‐CD47 (added for 1 or 3 days) or anti‐MHC‐I (added for 3 days) antibodies on cell viability was determined by PrestoBlue staining. (f) Number of propidium iodide‐positive C2C12 cells per field of view in the absence or presence of anti‐CD47 antibodies. Asterisks denote statistically significant differences from the control (*p < 0.05, Student's *t*‐test). *n* = at least 4 samples. Data are expressed as mean and individual values. ns: non‐significant.

To define this function, the effect of a neutralizing anti‐CD47 antibody on myoblast fusion and differentiation was tested. Neutralizing antibodies were shown to mimic the in vivo consequences of the Cd47 gene deletion in mice [30]. Our results demonstrate that C2C12 cells grown in the presence of anti‐CD47 antibodies for 3 days after the third day of differentiation have reduced fusion capacity (Figure 3a,b). Decreasing the time of antibody addition to the fifth day reduced the degree of fusion inhibition (Figure 3b). To exclude the possibility that anti‐CD47 antibodies impaired cell fusion by directly blocking the cell membrane alignment, we repeated the experiment with anti‐MHC‐I antibodies. Class I H2‐D1 and H2‐K1 MHC molecules are also implicated as “don't eat‐me” signals [19], and they show similar gene expression levels as Cd47 and Sirpα in C2C12 cells, but their receptor (PIR‐B) is not expressed in myoblasts (Figure 1d). Based on our results presented, anti‐MHC‐I antibodies did not interfere with cell fusion (Figure 3a,b). To exclude the possibility that anti‐CD47 antibody treatment impaired cell fusion by inhibiting myoblast differentiation, we measured the expression of differentiation markers in the presence and absence of anti‐CD47 antibodies. However, MyoD1, myogenin, myosin heavy chain (MYH) I, myomaker, and MYH4 expressions were not affected by the anti‐CD47 antibody treatment detected on the 6th day of myoblast differentiation (Figure 3c,d). Neither did we observe a decrease in the cell viability of anti‐CD47 antibody‐treated cultures, testing the viability of attached cells (Figure 3e), though the number of PI‐positive non‐attached cells increased (Figure 3f).

These results suggest that antibody targeting of cell surface CD47 molecules specifically interferes with cell fusion. However, based on these experiments, we could not decide whether CD47/SIRPα signaling is required for membrane fusion or, in its absence, myoblasts select the efferocytosis pathway.

### 3.4 Anti‐CD47 Antibody Treatment Increases Viable Red Blood Cell Uptake by Proliferating C2C12 Myoblasts but Does Not Affect the Apoptotic Thymocyte Uptake

**FIGURE 4:**
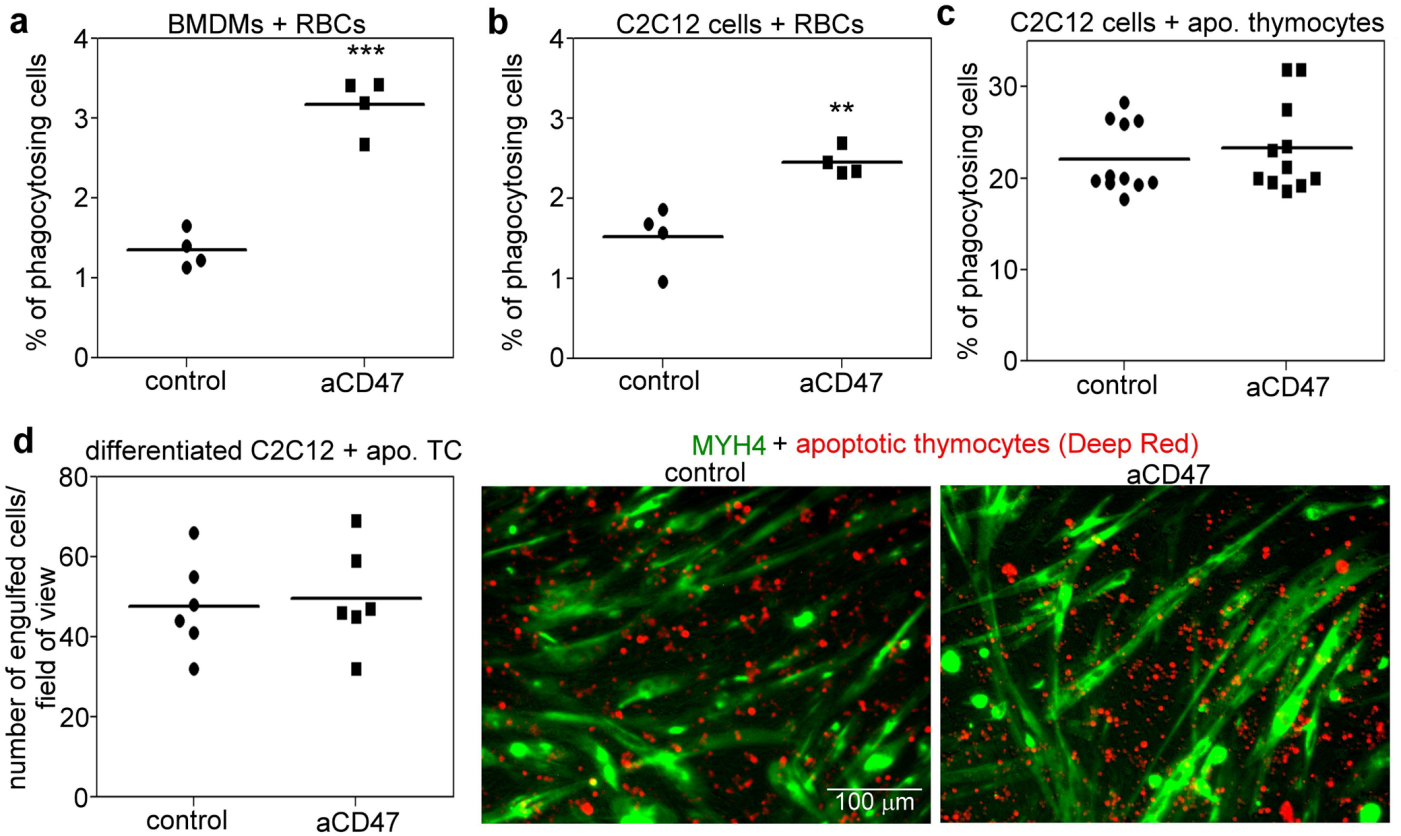
Viable red blood cell but not apoptotic thymocyte cell phagocytosis is enhanced by C2C12 cells in the presence of anti‐CD47 antibodies. (a) BMDMs were incubated with pHrodo green‐stained viable RBCs for 1 h in the absence or presence of anti‐CD47 antibodies. *n* = 4 (b) Proliferating C2C12 cells were incubated with pHrodo green‐stained viable RBCs for 1 h in the absence or presence of anti‐CD47 antibodies. (c) Proliferating C2C12 cells were incubated with Deep Red dye‐stained apoptotic thymocytes for 1 h in the absence or presence of anti‐CD47 antibodies. (d) The left panel shows the number of engulfed thymocytes within the C2C12 myotubes counted per field of view. The right panel shows representative fluorescent microscopic pictures of myosin heavy chain 4‐stained green myotubes containing engulfed red‐colored thymocytes *n* = at least 4. Asterisks denote statistically significant differences from the control (**p < 0.01, ***p < 0.001, Student's *t*‐test). Data are expressed as mean and individual values.

To answer the above question, first, we decided to answer whether CD47 signaling affects the uptake of viable cells by myoblasts. Targeting cell surface CD47 on the viable cells by neutralizing antibodies increases the uptake of viable cells by macrophages [17, 31]. To test the effect of anti‐CD47 antibody on viable cell engulfment by proliferating myoblasts, we isolated red blood cells (RBCs), and labeled them with pHrodo green dye, followed by incubation for 1 h with proliferating C2C12 cells or, as a control, with bone marrow‐derived macrophages (BMDMs). The anti‐CD47 antibody treatment enhanced RBC phagocytosis by both BMDMs (Figure 4a) and C2C12 cells (Figure 4b). Since, on apoptotic cells, the clustering formation of CD47 and consequently its avidity to SIRPα are lost, thus CD47 signaling does not influence apoptotic cell efferocytosis [32], the RBCs taken up must have been viable RBCs. In line with this, anti‐CD47 antibody treatment did not affect the uptake of apoptotic thymocytes by proliferating myoblasts (Figure 4c). Altogether, these data indicate that the CD47/SIRPα signaling pathway serves as a “don't eat‐me” signaling pathway in myoblasts as well.

While in the classical efferocytosis process, CD47 and SIRPα are engaged in the phagocytic synapse [33], in the myoblast confluent cultures, neighboring myoblasts might also interact via CD47 and SIRPα. That is why, we tested whether a potential inter‐myoblast signaling affects the efferocytosis process by comparing the apoptotic thymocyte uptake by confluent fusing myoblasts in the presence and absence of the anti‐CD47 antibodies. As shown in Figure 4d, confluent myoblast cultures were also effective in taking up apoptotic cells. However, anti‐CD47 antibody exposure did not influence the engulfment rate.

### 3.5 Long‐Term Exposure to Apoptotic Cells Alters Myoblast Fusion via Inhibiting PIEZO1

**FIGURE 5:**
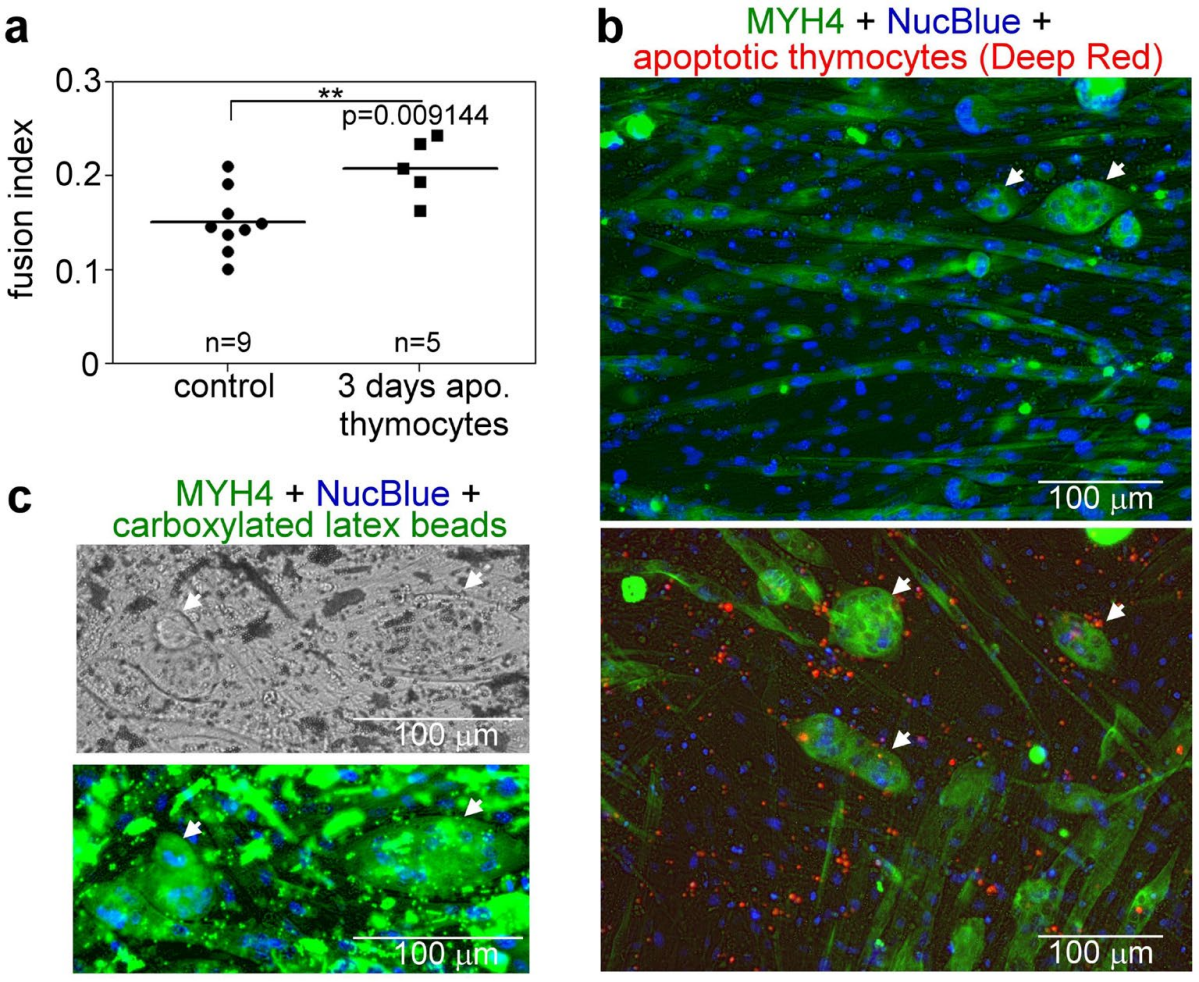
Exposure to apoptotic thymocytes enhances C2C12 myoblast fusion and results in syncytia formation. (a) Fusion indexes of 6‐day differentiated C2C12 cell cultures incubated in the absence or presence of apoptotic thymocytes for the last three days of their differentiation. Data are expressed as mean and individual values. (**p < 0.01, Student's *t*‐test). *n* = at least 5 samples. (b) Representative immunofluorescence images of myosin heavy chain 4 (green) and NucBlue (blue)‐stained 6‐day differentiated C2C12 cells forming multinucleated myotubes and oval‐shaped syncytia exposed to Deep Red‐stained apoptotic thymocytes for the 3 days of their differentiation. (c) Representative bright field and immunofluorescence images of myosin heavy chain 4 (green) and NucBlue (blue) stained 6‐day differentiated C2C12 cells forming multinucleated myotubes and syncytia in the presence of carboxylated latex beads administered during the last 3 days of their differentiation. White arrows in b and c indicate the formed abnormal syncytia.

Previous studies have shown that the presence of apoptotic cells enhanced the fusion of myoblasts [12]. After prolonged exposure of myoblasts to apoptotic thymocytes, we could confirm this finding (Figure 5a). Surprisingly, we also found that prolonged exposure of apoptotic cells to fusing myoblasts promoted the formation of oval‐shaped syncytia rather that of long myofibers (Figure 5b). Exposure to negatively charged carboxylated beads that mimic PS‐exposing apoptotic cells, and can be used as surrogate apoptotic cells in engulfment studies [34], had similar effects (Figure 5c). These findings indicate that constant PS exposure rather than the apoptotic cell content or molecules released from the apoptotic cells are inducers of the observed effect.

**FIGURE 6:**
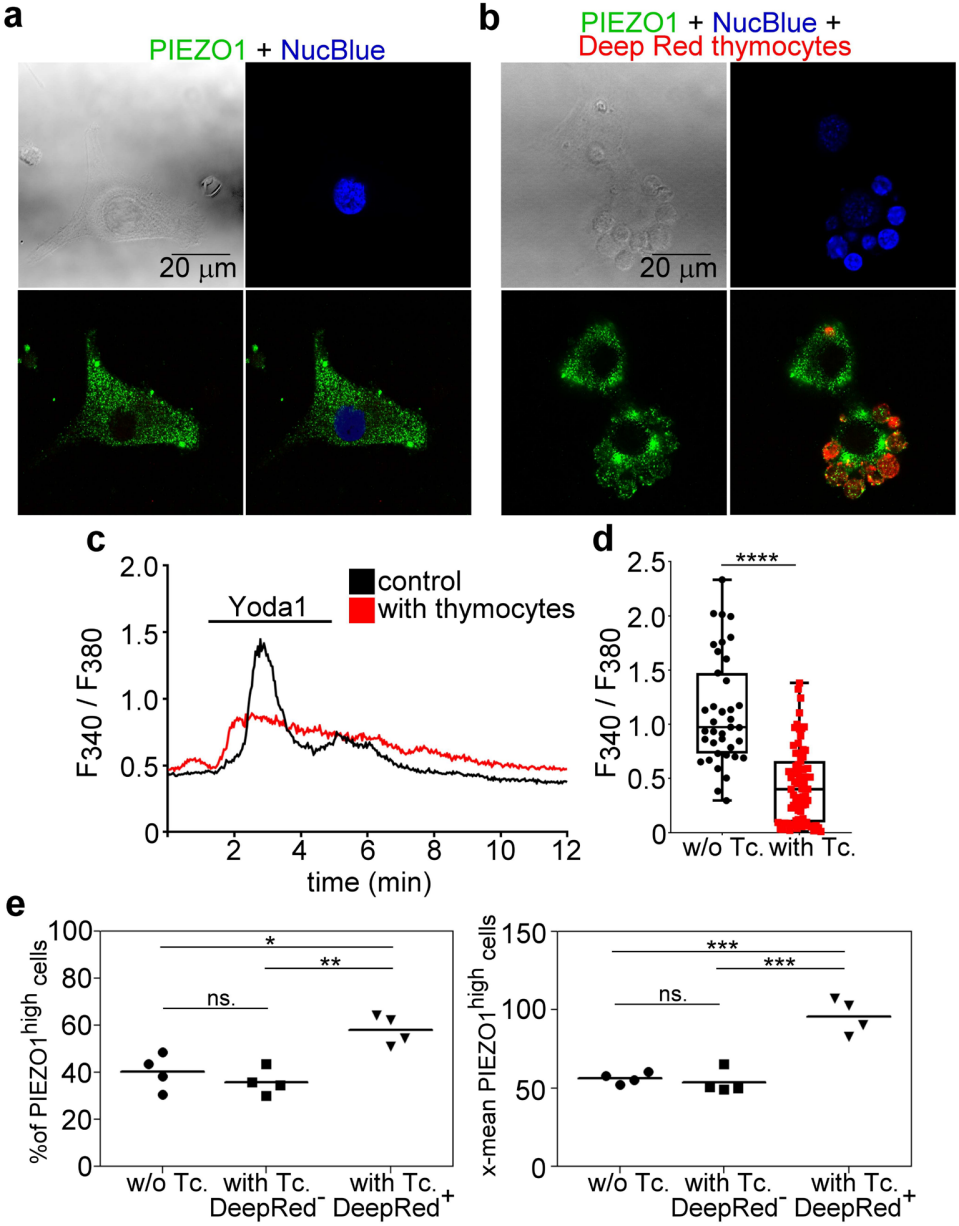
Myeloblast PIEZO1 channels are concentrated around the apoptotic cells and are inhibited by their presence. (a) Representative immunofluorescence images of PIEZO1 (green) and DAPI (blue)‐stained proliferating myoblasts. (b) Representative immunofluorescence images of PIEZO1 (green) and DAPI (blue)‐stained proliferating myoblasts in the presence of Deep Red‐stained apoptotic thymocytes. (c) Representative registrations of Yoda1 (25 μM)‐ induced changes in the cytosolic Ca^2+^ levels of proliferating C2C12 myoblasts in the presence and absence of apoptotic thymocytes. Cells were exposed to Yoda‐1 for the indicated time period. (d) Amplitudes of the Yoda1 (25 μM)‐induced calcium transients in the presence and absence of apoptotic thymocytes. (e) The percentage of PIEZO1^high^ control and apoptotic thymocytes‐exposed C2C12 cells (left panel) and their mean fluorescent intensity (right panel) determined by flow cytometry. Asterisk denotes statistically significant differences (*p < 0.05, **p < 0.01, ***p < 0.001, ****p < 0.0001, one‐way ANOVA with post hoc Tukey test). Data are expressed as mean and individual values. ns, non‐significant; Tc, thymocytes.

Since previous studies have demonstrated that constant expression of PS on fusing myoblasts leading to PIEZO1 inactivation results in syncytia formation [9], we decided to test whether exposure to apoptotic cells that express PS constantly also inhibits PIEZO1. First, we visualized the expression of PIEZO1 on myoblasts in the presence and absence of apoptotic cells, and found that apoptotic cells interacting with the myoblasts induced the concentration of cell surface PIEZO1 around the apoptotic cells (Figure 6a,b). For detecting PIEZO1 channel activity, the cytosolic Ca^2+^ concentrations were determined in C2C12 cells triggered by Yoda1, a PIEZO1 activator [35]. As seen in Figure 6c,d, interaction with apoptotic cells strongly inhibited the rise in the cytosolic Ca^2+^ concentrations of the myoblasts triggered by Yoda1. To rule out that the diminished Ca^2+^ signal is a consequence of a decreased ion channel protein expression, we determined cell surface PIEZO1 expression by flow cytometry in control and Deep Red dye‐labeled apoptotic thymocyte‐exposed C2C12 cells. We found that the percentage of PIEZO1^high^ cells and their mean fluorescence were significantly increased in the Deep Red positive, phagocytosing C2C12 cells as compared to control and to non‐phagocytosing cells (Figure 6e). The enhanced expression of PIEZO1 detected on the surface of phagocytosing cells was not related to bound apoptotic thymocytes, since apoptotic thymocytes express negligible amount of PIEZO1 as compared to myoblasts (Figure S1). These observations indicate that long‐term exposure to apoptotic cells induces abnormally shaped syncytia formation by fusing myoblasts, very likely via inhibiting PIEZO1 activity by the constantly expressed PS.

### 3.6 Anti‐CD47 Antibody Treatment Does Not Induce a Significant Increase in the Uptake of Viable Myoblasts

**FIGURE 7:**
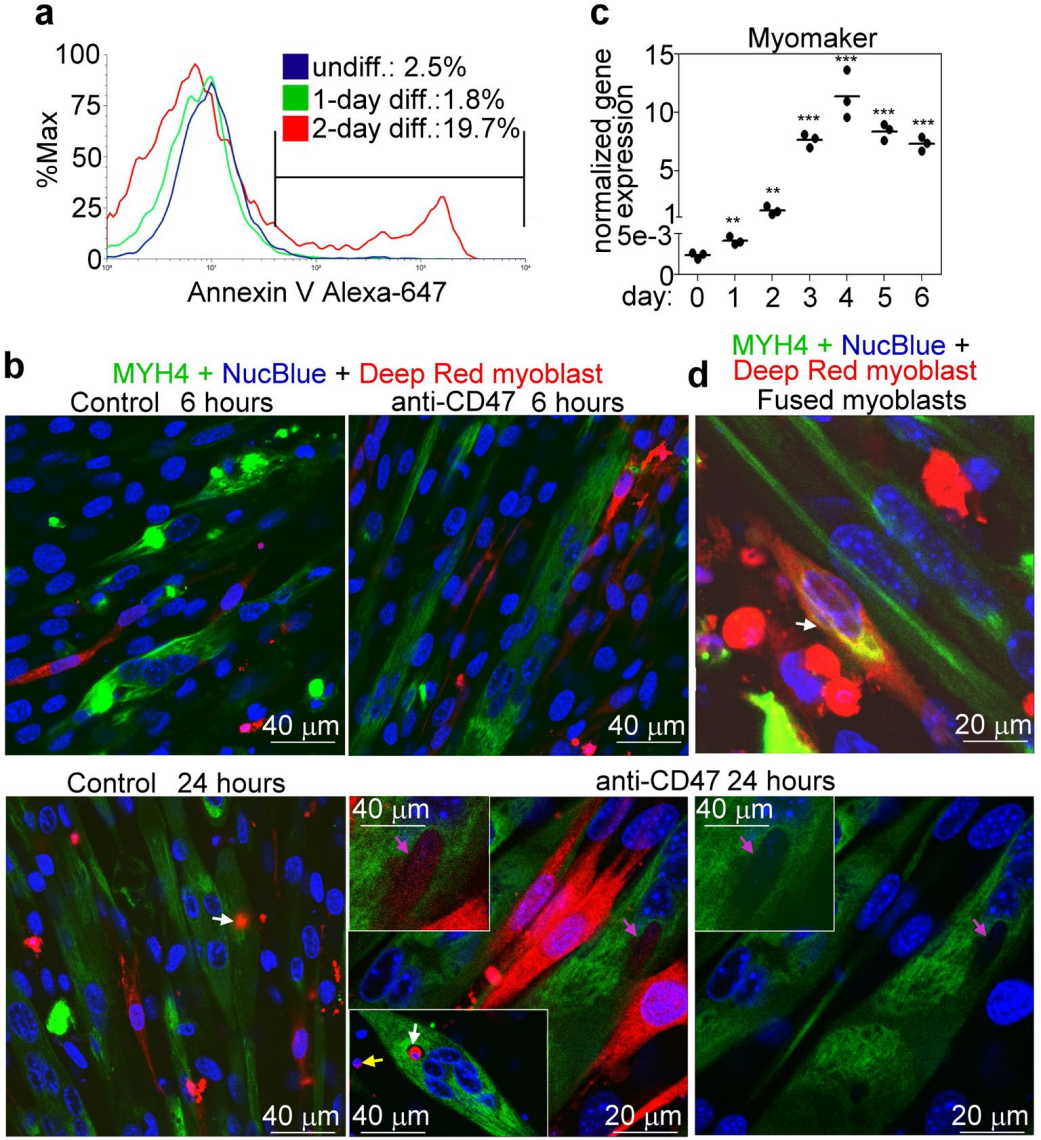
C2C12 myotubes can phagocytose neighboring cells. (a) Annexin V staining of undifferentiated, 1‐ or 2‐day differentiated C2C12 cells to detect the percentage of PS‐expressing myoblasts. (b) Confocal microscopic images of 6‐day differentiated C2C12 (myosin heavy chain 4‐stained green) cells incubated together with day 1 differentiated Deep Red‐stained C2C12 myoblasts for 6 or 24 h in the presence or absence of anti‐CD47 antibodies. The pink arrow indicates a viable myoblast engulfed by fusing myoblasts. The white arrows indicate engulfed apoptotic myoblasts inside the myotubes. The yellow arrow in the inset picture indicates an apoptotic myoblast that has not been engulfed yet. (c) Myomaker expression in differentiating C2C12 cells detected by qRT‐PCR. Data are expressed as mean and individual values. (**p < 0.01, ***p < 0.001, Student's *t*‐test). *n* = 3 samples. (d) Immunohistological staining of C2C12 cells fusion. C2C12 myoblasts were differentiated in 8‐well Ibidi chamber slides for 5 days and mixed with 3‐day differentiated, Deep Red‐stained myoblasts. After 3 h, the cells were stained for myosin heavy chain 4 and DNA, and confocal images were taken. The white arrow indicates a fused cell containing two nuclei and yellow cytosol due to the mixing of the red and green proteins originating from the two different fused cells.

Next, we tried to test whether viable cell engulfment occurs in fusing myoblast cultures that could explain the decrease in the myoblast fusion induced by anti‐CD47 antibodies. Since these cultures cannot be analyzed by FACS, we decided to test by adding day 1 differentiated viable myoblasts, which were reported to start expressing PS [12], to fusing myoblasts, whether fusing myoblasts are capable of engulfing them in the presence and absence of CD47 signaling. For this purpose, single myoblast cells were stained with Deep Red, while fusing myoblasts with an Alexa Fluor 488‐labeled antibody for MYH4, and we were searching for red myoblasts within the already fused cells. Since 1‐day differentiated myoblasts are not fusion competent yet, their appearance within the fusing myoblasts could be the result of efferocytosis only. As seen in Figure 7b, 1‐day differentiated myoblasts did not, but 2‐day differentiated myoblasts started to express PS detected by Annexin V labeling. The pictures in Figure 7c demonstrate that inhibition of CD47 signaling did not promote the uptake of viable myoblasts at 6 h, either because they did not express PS, or the time was too short for detectable cell uptake. However, after 24 h of coculture, we observed engulfment of viable myoblasts, though as a rare event (1–2 engulfed cells/100 nuclei), which was not observed in the control cultures. As proof of engulfment, we also visualized myoblast fusion by administering red color‐stained 3‐day differentiated myoblasts to 5‐day differentiated green MYH4‐stained myoblasts. The 3‐day differentiated cells already express abundantly the fusion protein myomaker and are capable of fusion (Figure 7d). While following viable cell uptake, the engulfed myoblasts kept their shape and color (Figure 7c pink arrows), the red and green proteins originating from two fused cells were mixed, appearing as yellow (Figure 7e).

On the other hand, uptake of apoptotic myoblasts, which are produced as a result of anoikis, was observed in both the control and the anti‐CD47‐treated cultures. The lower inset demonstrates that the apoptotic cells were formed outside of the myoblasts, not inside from the engulfed living cells. Altogether, our data demonstrate that, when CD47 signaling is inhibited during myoblast fusion, engulfment of viable myoblasts can occur, but the frequency of it is too low to explain the observed degree of cell fusion inhibition.

### 3.7 Ligation of CD47 by Thrombospondin‐1 Might Also Contribute to the Fusion of the Myoblasts

**FIGURE 8:**
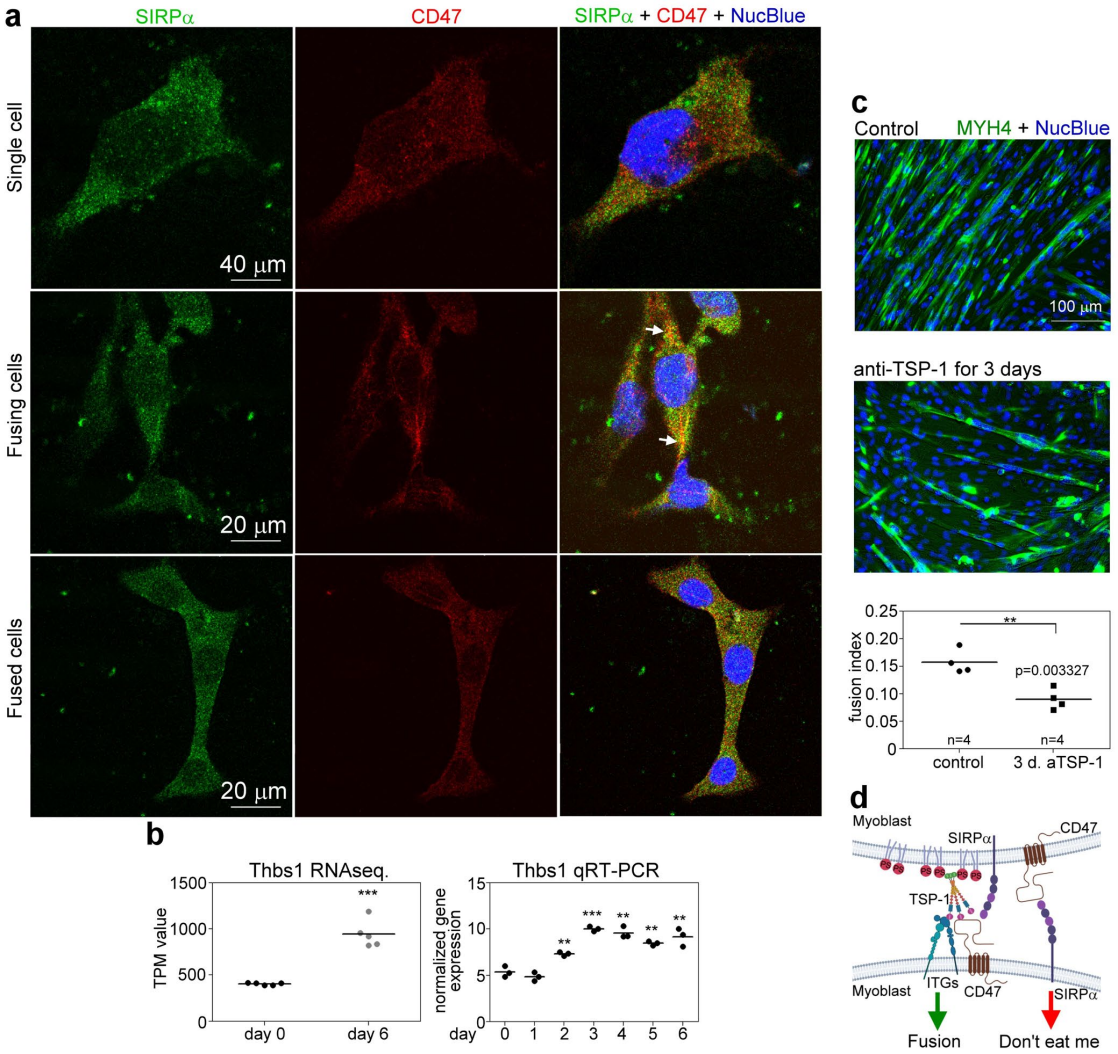
CD47, SIRPα, and TSP‐1 are required for proper myoblast fusion. (a) Confocal microscopic images of 3‐day differentiated C2C12 cells stained for CD47 (red) and SIRPα (green) proteins, and DNA (blue), demonstrating accumulation of both CD47 and SIRPα in the finger‐like myoblast filopodia at the contact site between the fusing cells. The white arrow indicates the contact site between neighboring myoblasts. (b) Left panel: the average TPM values for Thbs1 in proliferating and 6‐day differentiated C2C12 cells detected by RNA sequencing. Right panel: the mRNA expression level of Thbs1 in proliferating and differentiating C2C12 cells determined by qRT‐PCR. Data are expressed as mean and individual values. (**p < 0.01, ***p < 0.001, Student's *t*‐test). *n* = 5 and *n* = 3 samples for RNA sequencing and qRT‐PCR, respectively. (c) Fusion indexes and representative immunofluorescence images of myosin heavy chain 4 (green) and NucBlue (blue)‐stained 6‐day differentiated C2C12 cells growing in the absence or presence of neutralizing anti‐TSP‐1 antibodies during the last three days of their differentiation. (**p < 0.01, Student's *t*‐test). *n* = 4 samples. (d) Proposed model for the involvement of CD47 in the regulation of myoblast fusion. CD47, acting in a cis‐manner with integrins, promotes cell fusion. TSP‐1 serves as a bridging molecule for these proteins to recognize PS on the surface of the other fusing myoblast [38]. In addition, CD47 acting in a trans‐manner activates SIRPα signaling to prevent accidental viable cell uptake.

Since SIRPα is a well‐established partner of CD47 in the “do not eat‐me” signaling pathway, and was suggested to participate in macrophage multinucleation [36] and osteoclast formation [37], two processes that also involve cell fusion, we decided to test whether SIRPα and CD47 colocalize during myoblast fusion. As seen in Figure 8a, while the two proteins were diffusively found on the surface of single and fused myoblasts, we observed accumulation of both CD47 and SIRPα in the finger‐like myoblast filopodia [39] at the contact site between the cells, further implying the involvement of the two proteins in the fusion process.

Since, in addition to SIRPα, thrombospondin‐1 (TSP‐1), a PS‐binding bridging molecule, was also reported to be a ligand for skeletal muscle CD47, and to affect skeletal muscle biology [40], we checked whether TSP‐1 is expressed by C2C12 cells. Turning again to the GSE215627 database, we found that proliferating C2C12 cells express Thbs1 mRNA, and its expression is increased significantly during C2C12 myoblast differentiation. These results were confirmed by us using qRT‐PCR (Figure 8b). Similar was the finding in vivo during skeletal muscle regeneration following cardiotoxin injury (Figure 2a). To decide whether TSP‐1 could be a ligand of CD47 in promoting myoblast fusion, the fusion of C2C12 cells was determined in the presence of a neutralizing TSP‐1 antibody, which was previously shown to interfere with the CD47 signaling [40]. As shown in Figure 8c, administration of this antibody on the 3rd, 4th, and 5th day of differentiation decreased the fusion of myoblasts, indicating that, in addition to SIRPα, TSP‐1 might also be a ligand of CD47 in the regulation of the myoblast fusion process (Figure 8d).

## 4 Discussion

Both efferocytosis and myoblast fusion are initiated by tight cellular membrane alignment of two interacting cells. Not surprisingly, both processes utilize the same receptors to facilitate this mechanism. From the apoptotic cell's or the fusing myoblast's part, PS appears on the cell surface in a caspase‐dependent manner [6, 9, 41]. However, while on the apoptotic cells the PS exposure is constant [41], during myoblast fusion the PS expression is transient [4, 5], and phospholipid flippase activation that translocates the cell surface‐exposed PS to the inner leaflet of the plasma membrane is required for proper myoblast fusion and myotube elongation [9]. Lack of flippase activity resulting in constant PS exposure severely interferes with the myotube formation process, leading to the generation of large syncytia [9].

PS receptors, which were first described as receptors promoting efferocytosis expressed by professional and non‐professional phagocytes, were found to be expressed by myoblasts as well, but to mediate primarily the myoblast fusion process [3, 8, 12, 13, 14, 15]. Interestingly, recently we found that the same receptors promoted efferocytosis by myoblasts as well [16]. The present paper addresses how myoblasts prevent accidental phagocytosis during myoblast fusion. Logically, we tested the possible involvement of “don't eat‐me” signals in the decision‐making. Indeed, we detected the expression of several “don't eat‐me” signal molecules in differentiating myoblasts, both in vitro and in vivo, from which the expression of the CD47/SIRPα pair was the highest. In addition, the expression of SIRPα significantly increased during myoblast differentiation, reaching its peak expression by the time myoblasts became fusion‐competent. Inhibition of CD47 by anti‐CD47 antibodies strongly reduced the fusion of the myoblasts without affecting their viability or differentiation. To decide whether the CD47/SIRPα signaling is required for fusion, or the fusion is reduced due to enhanced viable cell uptake, we tested viable cell uptake by myoblasts in the presence of anti‐CD47 antibodies. We found that these antibodies promoted viable red blood cell efferocytosis by proliferating myoblasts, indicating that CD47 serves as a “don't‐eat‐me” signal in myoblasts as well.

As expected, CD47 did not affect the engulfment of apoptotic cells either by proliferating or by fusing myoblasts. Surprisingly, however, prolonged exposure to apoptotic cells interfered with the myoblast tube formation process, leading to the accumulation of large syncytia. It is very likely that the constant exposure of PS on the surface of apoptotic cells, which, as we have shown, inhibited PIEZO1 activity, is responsible for the phenomenon, since loss of PIEZO1 activity was reported to result in syncytia formation by fusing myoblasts [9]. PIEZO1 activation initiates actomyosin fiber assembly beneath the plasma membrane to prevent uncontrolled fusion [9]. Thus, its inhibition by PS of the apoptotic cells might also explain the previously reported enhanced myoblast fusion in the presence of apoptotic cells [12], which we could also confirm.

PIEZO1 is involved in the regulation of various mechanosensation‐based biological processes [42], including the regulation of bone homeostasis by being expressed on osteoblastic cells [43]. PIEZO1 deficiency in osteoblastic cells leads to loss of bone mass and spontaneous fractures with increased bone resorption [43], a phenomenon found in many patients undergoing hematopoietic stem cell therapy [44], which includes irradiation and consequent apoptosis of all the bone marrow‐derived cells, and for which the pathologic reason has not been found so far. Similarly, PIEZO1 was shown to be involved in jawbone development [45] and in alveolar bone remodeling [46]. Accumulation of apoptotic cells has been associated with the severity of periodontitis [47], and the worst outcome of periodontitis is also bone resorption [48]. PIEZO1 protein contains a site for PS binding [49], thus, inhibition of PIEZO1 activity by constantly expressed PS on apoptotic cells is very likely not specific for myoblasts. Based on our data, we propose that accumulating apoptotic cells in the bone marrow following irradiation or in the periodontic tissues during chronic inflammation might interfere with PIEZO1 signaling in the osteoblastic cells, contributing to the observed enhanced bone resorption. Thus, it would be worth testing the involvement of PIEZO1 and the effect of PIEZO1‐activating molecules in these diseases to combat loss of the healthy bone structure. What is more, it is interesting to speculate that in the case of any pathological situation, which leads to the accumulation of apoptotic cells, the function of PIEZO1 of neighboring cells can be affected and might contribute to the clinical features of the disease. In terms of skeletal muscle regeneration, extensive muscle death is a hallmark of several muscle diseases, such as dystrophies, inflammatory myopathies, metabolic and mitochondrial myopathies, and can lead to continuous production of PS‐expressing dead myofibers, stromal cells, and tissue‐infiltrating inflammatory leukocytes, which can potentially regulate PIEZO1 activity in the neighboring muscle cells [50]. Indeed, in muscle transplantation experiments, grafted donor muscle cells showed enhanced fusion with the dystrophic muscles of mdx mice [51].

Though interfering with the CD47 signaling promoted viable red blood cell uptake, it did not result in such a significant induction in the uptake of viable myoblasts by fusing myoblasts that could explain the strong inhibition of myoblast fusion that we observed, indicating that CD47 might also be required for the fusion process itself. Indeed, CD47, as a SIRPα partner, has already been suggested to participate in the macrophage multinucleation [36] and in the multinucleated osteoclast formation [37], processes that also involve cell fusion. Myoblast fusion was shown to take place through filopodia, finger‐like protrusions on lamellipodial extensions of the cells [39]. Using confocal microscopy, we could detect both CD47 and SIRPα accumulation in these lamellipodial extensions at the contact sites between neighboring myoblasts. In addition, we also demonstrated that another ligand of CD47, TSP‐1, a PS‐binding bridging molecule, is also produced by myoblasts and contributes to cell fusion. It must be noted, however, that TSP‐1, in addition to being a CD47 ligand, is a complex multi‐domain matricellular protein that mediates various other cell–cell and cell‐matrix interactions [52]. Thus, TSP‐1 is known to bind β3 and β1 integrins, as well as the scavenger receptor CD36, independently of CD47, via its type II and III repeats, while its other domains bind collagens, proteases, and latent growth factors. These multifunctional features enable TSP‐1 to promote cell adhesion and signaling through integrins and other receptors, all of which might contribute to myoblast fusion. Thus, the functional roles of TSP‐1 in fusion likely reflect a combination of CD47‐dependent and CD47‐independent mechanisms.

CD47 was discovered as an integrin‐associated protein that can form a functional G protein‐activating receptor with various integrins [53] from which several were shown to contribute to the myoblast fusion process [8]. In addition to CD47, two further integrin coreceptors, CD36 [54] and transglutaminase 2 [15], were also reported to participate in the myoblast fusion. These coreceptors also use bridging molecules to bind to PS [55]. Thus, integrins, their coreceptors CD36, CD47, and TG2, together with their bridging molecules, might form a tight bond with the PS‐expressing myoblast to promote lamellopodial extensions and stabilize cell membranes for fusion.

Recently, muscle‐specific CD47 knockout mice have been generated [30]. However, the direct involvement of CD47 in myoblast fusion would be difficult to test in vivo in these mice, as CD47 also regulates the proliferation of muscle stem cells [40] as well as the metabolism of skeletal muscle [30]. Altogether, our data demonstrate that differentiating myoblasts upregulate “don't eat‐me” signals and their receptors, likely to avoid accidental engulfment of the fusing cells, and, surprisingly, also to promote myotube formation.

## Author Contributions

Conceptualization was carried out by Zsolt Sarang and Zsuzsa Szondy. Visualization of data was carried out by Zsolt Sarang. The manuscript was written by Zsuzsa Szondy. Experiments and data analysis were carried out by Maysaa Adil Ali, Albert Bálint Papp, Éva Garabuczi, Áron Károly Gere, Beatrix Dienes, Mónika Gönczi, Albert Bálint Papp, György Vámosi, Krisztina Köröskényi, László Csernoch, Zsuzsa Szondy. All authors approved the submission of the present version of the manuscript.

## Funding

This work was supported by the National Research, Development, and Innovation Office (138162, 137600, and ANN 135107), Project No. TKP2021‐EGA‐18 (under the TKP2021‐EGA funding scheme), the Stipendium Hungaricum Scholarship, and the Research Funding System of the Medical Faculty of the University of Debrecen.

## Conflicts of Interest

The authors declare no conflicts of interest.

## Supporting information


**Figure S1:** Gating strategy for cell surface detection of PIEZO1 on C2C12 cells and on thymocytes. The two cell types were separated based on their marked differences in light‐scattering properties. Phagocytosing C2C12 cells were gated based on their Deep Red dye positivity and further analyzed for PIEZO1 expression.

## Data Availability

The RNA sequencing data that support the findings of this study are openly available in the Gene Expression Omnibus repository at https://www.ncbi.nlm.nih.gov/geo/, reference number GSE215627 (DOI: 10.3390/cells11223549). Other data are available from the corresponding author upon reasonable request.

## References

[1] T. Braun and M. Gautel , “Transcriptional Mechanisms Regulating Skeletal Muscle Differentiation, Growth and Homeostasis,” Nature Reviews. Molecular Cell Biology 12 (2011): 349–361. 21602905 10.1038/nrm3118

[2] R. J. Bryson‐Richardson and P. D. Currie , “The Genetics of Vertebrate Myogenesis,” Nature Reviews. Genetics 9 (2008): 632–646. 10.1038/nrg2369 18636072

[3] S. M. Abmayr and G. K. Pavlath , “Myoblast Fusion: Lessons From Flies and Mice,” Development 139 (2012): 641–656. 22274696 10.1242/dev.068353 PMC3265056

[4] S. M. van den Eijnde , M. J. B. Hoff , C. P. M. Reutelingsperger , et al., “Transient Expression of Phosphatidylserine at Cell‐Cell Contact Areas Is Required for Myotube Formation,” Journal of Cell Science 114 (2001): 3631–3642. 11707515 10.1242/jcs.114.20.3631

[5] J. Jeong and I. M. Conboy , “Phosphatidylserine Directly and Positively Regulates Fusion of Myoblasts Into Myotubes,” Biochemical and Biophysical Research Communications 414 (2011): 9–13. 21910971 10.1016/j.bbrc.2011.08.128 PMC3195849

[6] M. H. Dehkordi , A. Tashakor , E. O'Connell , and H. O. Fearnhead , “Apoptosome‐Dependent Myotube Formation Involves Activation of Caspase‐3 in Differentiating Myoblasts,” Cell Death & Disease 11 (2020): 308, 10.1038/s41419-020-2502-4. 32366831 PMC7198528

[7] E. Leikina , D. G. Gamage , V. Prasad , et al., “Myomaker and Myomerger Work Independently to Control Distinct Steps of Membrane Remodeling During Myoblast Fusion,” Developmental Cell 46 (2018): 767–780.e7. 30197239 10.1016/j.devcel.2018.08.006 PMC6203449

[8] Z. Szondy , N. Al‐Zaeed , N. Tarban , E. Fige , E. Garabuczi , and Z. Sarang , “Involvement of Phosphatidylserine Receptors in Skeletal Muscle Regeneration: Therapeutic Implications,” Journal of Cachexia, Sarcopenia and Muscle 13 (2022): 1961–1973. 35666022 10.1002/jcsm.13024 PMC9397555

[9] M. Tsuchiya , Y. Hara , M. Okuda , et al., “Cell Surface Flip‐Flop of Phosphatidylserine Is Critical for PIEZO1‐Mediated Myotube Formation,” Nature Communications 9 (2018): 2049, 10.1038/s41467-018-04436-w. PMC5967302 29799007

[10] V. Depraetere , “‘Eat Me’ Signals of Apoptotic Bodies,” Nature Cell Biology 2 (2000): E104, 10.1038/35014098. 10854338

[11] S. Arandjelovic and K. S. Ravichandran , “Phagocytosis of Apoptotic Cells in Homeostasis,” Nature Immunology 16 (2015): 907–917. 26287597 10.1038/ni.3253 PMC4826466

[12] A. E. Hochreiter‐Hufford , C. S. Lee , J. M. Kinchen , et al., “Phosphatidylserine Receptor BAI1 and Apoptotic Cells as New Promoters of Myoblast Fusion,” Nature 497 (2013): 263–267. 23615608 10.1038/nature12135 PMC3773542

[13] S. Y. Park , Y. Yun , J. S. Lim , et al., “Stabilin‐2 Modulates the Efficiency of Myoblast Fusion During Myogenic Differentiation and Muscle Regeneration,” Nature Communications 7, no. 1 (2016): 10871, 10.1038/ncomms10871. PMC4793076 26972991

[14] N. Al‐Zaeed , Z. Budai , Z. Szondy , and Z. Sarang , “TAM Kinase Signaling Is Indispensable for Proper Skeletal Muscle Regeneration in Mice,” Cell Death & Disease 12 (2021): 611, 10.1038/s41419-021-03892-5. 34120143 PMC8197762

[15] Z. Budai , N. Al‐Zaeed , P. Szentesi , et al., “Impaired Skeletal Muscle Development and Regeneration in Transglutaminase 2 Knockout Mice,” Cells 10 (2021): 3089, 10.3390/cells10113089. 34831312 PMC8623654

[16] M. Adil Ali , É. Garabuczi , N. Tarban , and Z. Sarang , “All‐Trans Retinoic Acid and Dexamethasone Regulate Phagocytosis‐Related Gene Expression and Enhance Dead Cell Uptake in C2C12 Myoblast Cells,” Scientific Reports 13 (2023): 21001, 10.1038/s41598-023-48492-9. 38017321 PMC10684882

[17] P. Burger , P. Hilarius‐Stokman , D. de Korte , T. K. van den Berg , and R. van Bruggen , “CD47 Functions as a Molecular Switch for Erythrocyte Phagocytosis,” Blood 119 (2012): 5512–5521. 22427202 10.1182/blood-2011-10-386805

[18] A. A. Barkal , R. E. Brewer , M. Markovic , et al., “CD24 Signalling Through Macrophage Siglec‐10 Is a Target for Cancer Immunotherapy,” Nature 572 (2019): 392–396. 31367043 10.1038/s41586-019-1456-0 PMC6697206

[19] A. A. Barkal , K. Weiskopf , K. S. Kao , et al., “Engagement of MHC Class I by the Inhibitory Receptor LILRB1 Suppresses Macrophages and Is a Target of Cancer Immunotherapy,” Nature Immunology 19 (2018): 76–84. 29180808 10.1038/s41590-017-0004-z PMC5832354

[20] V. R. Simhadri , J. F. Andersen , E. Calvo , S. C. Choi , J. E. Coligan , and F. Borrego , “Human CD300a Binds to Phosphatidylethanolamine and Phosphatidylserine, and Modulates the Phagocytosis of Dead Cells,” Blood 119 (2012): 2799–2809. 22302738 10.1182/blood-2011-08-372425 PMC3327458

[21] S. Brown , I. Heinisch , E. Ross , K. Shaw , C. D. Buckley , and J. Savill , “Apoptosis Disables CD31‐Mediated Cell Detachment From Phagocytes Promoting Binding and Engulfment,” Nature 418 (2002): 200–203. 12110892 10.1038/nature00811

[22] S. M. Kelley and K. S. Ravichandran , “Putting a Brake on Phagocytosis: “Don't‐Eat‐Me” Signaling in Physiology and Disease,” EMBO Reports 22 (2021): e52564, 10.15252/embr.202152564. 34041845 PMC8183410

[23] H. Deng , G. Wang , S. Zhao , et al., “New Hope for Tumor Immunotherapy: The Macrophage‐Related ‘Do Not Eat Me’ Signaling Pathway,” Frontiers in Pharmacology 14 (2023): 1228962, 10.3389/fphar.2023.1228962. 37484024 PMC10358856

[24] P. Lyu and H. Jiang , “RNA‐Sequencing Reveals Upregulation and a Beneficial Role of Autophagy in Myoblast Differentiation and Fusion,” Cells 11 (2022): 3549, 10.3390/cells11223549. 36428978 PMC9688917

[25] A. Patsalos , P. Tzerpos , L. Halasz , et al., “The BACH1‐HMOX1 Regulatory Axis Is Indispensable for Proper Macrophage Subtype Specification and Skeletal Muscle Regeneration,” Journal of Immunology 203 (2019): 1532–1547. 10.4049/jimmunol.1900553 PMC6736746 31405954

[26] K. Köröskényi , E. Duró , A. Pallai , et al., “Involvement of Adenosine A 2A Receptors in Engulfment‐Dependent Apoptotic Cell Suppression of Inflammation,” Journal of Immunology 186 (2011): 7144–7155. 10.4049/jimmunol.1002284 PMC3395167 21593381

[27] D. Yaffe and O. Saxel , “Serial Passaging and Differentiation of Myogenic Cells Isolated From Dystrophic Mouse Muscle,” Nature 270 (1977): 725–727. 563524 10.1038/270725a0

[28] Y. Wang , J. Lu , and Y. Liu , “Skeletal Muscle Regeneration in Cardiotoxin‐Induced Muscle Injury Models,” International Journal of Molecular Sciences 23 (2022): 13380, 10.3390/ijms232113380. 36362166 PMC9657523

[29] N. Tarban , A. B. Papp , D. Deák , et al., “Loss of Adenosine A3 Receptors Accelerates Skeletal Muscle Regeneration in Mice Following Cardiotoxin‐Induced Injury,” Cell Death & Disease 14 (2023): 706, 10.1038/s41419-023-06228-7. 37898628 PMC10613231

[30] Y. Su , J. Sun , X. Li , et al., “CD47‐Blocking Antibody Confers Metabolic Benefits Against Obesity,” Cell Reports Medicine 6, no. 102089 (2025), 10.1016/j.xcrm.2025.102089. PMC12147847 40267910

[31] S. J. Gardai , K. McPhillips , S. C. Frasch , et al., “Cell‐Surface Calreticulin Initiates Clearance of Viable or Apoptotic Cells Through Trans‐Activation of LRP on the Phagocyte,” Cell 123 (2005): 321–334. 16239148 10.1016/j.cell.2005.08.032

[32] Z. Lv , Z. Bian , L. Shi , et al., “Loss of Cell Surface CD47 Clustering Formation and Binding Avidity to SIRPα Facilitate Apoptotic Cell Clearance by Macrophages,” Journal of Immunology 195 (2015): 661–671. 10.4049/jimmunol.1401719 PMC4490976 26085683

[33] R. K. Tsai and D. E. Discher , “Inhibition of “Self” Engulfment Through Deactivation of Myosin‐II at the Phagocytic Synapse Between Human Cells,” Journal of Cell Biology 180 (2008): 989–1003, 10.1083/jcb.200708043. 18332220 PMC2265407

[34] B. Tóth , E. Garabuczi , Z. Sarang , et al., “Transglutaminase 2 Is Needed for the Formation of an Efficient Phagocyte Portal in Macrophages Engulfing Apoptotic Cells,” Journal of Immunology 182 (2009): 2084–2092. 10.4049/jimmunol.0803444 19201861

[35] W. M. Botello‐Smith , W. Jiang , H. Zhang , et al., “A Mechanism for the Activation of the Mechanosensitive Piezo1 Channel by the Small Molecule Yoda1,” Nature Communications 10 (2019): 4503, 10.1038/s41467-019-12501-1. PMC6776524 31582801

[36] X. Han , H. Sterling , Y. Chen , et al., “CD47, a Ligand for the Macrophage Fusion Receptor, Participates in Macrophage Multinucleation,” Journal of Biological Chemistry 275 (2000): 37984–37992. 10964914 10.1074/jbc.M002334200

[37] P. Lundberg , C. Koskinen , P. A. Baldock , et al., “Osteoclast Formation Is Strongly Reduced Both In Vivo and In Vitro in the Absence of CD47/SIRPα‐Interaction,” Biochemical and Biophysical Research Communications 12 (2007): 444–448. 10.1016/j.bbrc.2006.11.057 17126807

[38] M. J. Calzada , D. S. Annis , B. Zeng , et al., “Identification of Novel β1 Integrin Binding Sites in the Type 1 and Type 2 Repeats of Thrombospondin‐1,” Journal of Biological Chemistry 279 (2004): 41734–41743. 15292271 10.1074/jbc.M406267200

[39] D. V. Hammers , C. C. Hart , M. K. Matheny , et al., “Filopodia Powered by Class × Myosin Promote Fusion of Mammalian Myoblasts,” eLife 10 (2021): e72419, 10.7554/eLife.72419. 34519272 PMC8500716

[40] E. Porpiglia , T. Mai , P. Kraft , et al., “Elevated CD47 Is a Hallmark of Dysfunctional, Aged Muscle Stem Cells That Can Be Targeted to Augment Regeneration,” Cell Stem Cell 29 (2022): 1653–1668.e8. 36384141 10.1016/j.stem.2022.10.009 PMC9746883

[41] S. Nagata and K. Segawa , “Sensing and Clearance of Apoptotic Cells,” Current Opinion in Immunology 68 (2021): 1–8, 10.1016/j.coi.2020.07.007. 32853880

[42] X.‐Z. Fang , T. Zhou , J. Q. Xu , et al., “Structure, Kinetic Properties and Biological Function of Mechanosensitive Piezo Channels,” Cell & Bioscience 11 (2021): 13, 10.1186/s13578-020-00522-z. 33422128 PMC7796548

[43] L. Wang , X. You , S. Lotinun , L. Zhang , N. Wu , and W. Zou , “Mechanical Sensing Protein PIEZO1 Regulates Bone Homeostasis via Osteoblast‐Osteoclast Crosstalk,” Nature Communications 11 (2020): 282, 10.1038/s41467-019-14146-6. PMC6962448 31941964

[44] X. N. Pundole , A. G. Barbo , H. Lin , R. E. Champlin , and H. Lu , “Increased Incidence of Fractures in Recipients of Hematopoietic Stem‐Cell Transplantation,” Journal of Clinical Oncology 33 (2015): 1364–1370. 25779562 10.1200/JCO.2014.57.8195 PMC5320933

[45] X. Nie , Y. Abbasi , and M. K. Chung , “Piezo1 and Piezo2 Collectively Regulate Jaw Development,” Development 151 (2024): dev202386, 10.1242/dev.202386. 38619396 PMC11128276

[46] Y. Du and K. Yang , “Role of Mechanosensitive Ion Channel Piezo1 in Tension‐Side Orthodontic Alveolar Bone Remodeling in Rats,” Archives of Oral Biology 155 (2023): 105798, 10.1016/j.archoralbio.2023.105798. 37651768

[47] B. Song , T. Zhou , W. L. Yang , J. Liu , and L. Q. Shao , “Programmed Cell Death in Periodontitis: Recent Advances and Future Perspectives,” Oral Diseases 23 (2017): 609–619. 27576069 10.1111/odi.12574

[48] S. A. Hienz , S. Paliwal , and S. Ivanovski , “Mechanisms of Bone Resorption in Periodontitis,” Journal of Immunology Research 2015 (2015): 615486, 10.1155/2015/615486. 26065002 PMC4433701

[49] A. Buyan , C. D. Cox , J. Rae , et al., “Piezo1 Induces Local Curvature in a Mammalian Membrane and Forms Specific Protein‐Lipid Interactions,” Preprint at *BioRxiv* (2019), 10.1101/787531.

[50] C. Sciorati , E. Rigamonti , A. A. Manfredi , and P. Rovere‐Querini , “Cell Death, Clearance and Immunity in the Skeletal Muscle,” Cell Death and Differentiation 23 (2016): 927–937. 26868912 10.1038/cdd.2015.171 PMC4987728

[51] G. M. Smythe , Y. Fan , and M. D. Grounds , “Enhanced Migration and Fusion of Donor Myoblasts in Dystrophic and Normal Host Muscle,” Muscle & Nerve 23 (2000): 560–574. 10716768 10.1002/(sici)1097-4598(200004)23:4<560::aid-mus16>3.0.co;2-g

[52] A. Kale , N. M. Rogers , and K. Ghimire , “Thrombospondin‐1 CD47 Signalling: From Mechanisms to Medicine,” International Journal of Molecular Sciences 22 (2021): 4062. 33920030 10.3390/ijms22084062 PMC8071034

[53] E. Brown , “Integrin‐Associated Protein (CD47): An Unusual Activator of G Protein Signaling,” Journal of Clinical Investigation 107 (2001): 1499–1500. 11413154 10.1172/JCI13315 PMC200200

[54] S. Y. Park , Y. Yun , and I. S. Kim , “CD36 Is Required for Myoblast Fusion During Myogenic Differentiation,” Biochemical and Biophysical Research Communications 427 (2012): 705–710. 23036201 10.1016/j.bbrc.2012.09.119

[55] M. Chikazawa , M. Shimizu , Y. Yamauchi , and R. Sato , “Bridging Molecules Are Secreted From the Skeletal Muscle and Potentially Regulate Muscle Differentiation,” Biochemical and Biophysical Research Communications 522 (2020): 113–120. 31753488 10.1016/j.bbrc.2019.11.010

