# Supplementary figures and images for: CD47 Promotes, While Exposure to Apoptotic Cells Destroys the Fusion Program of Differentiating Myoblasts
Source: FASEB J. 2026 Feb 12;40(4):e71578. doi: 10.1096/fj.202503809R (PMC12897927; doi:10.1096/fj.202503809R)

Supplementary figure 1.jpg

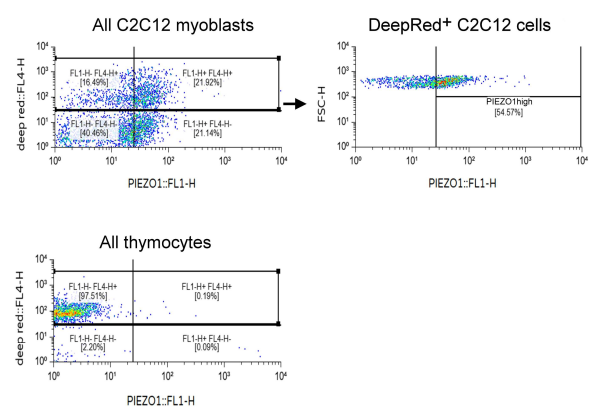

Supplement: Supplementary file 1 — Figure S1: Gating strategy for cell surface detection of PIEZO1 on C2C12 cells and on thymocytes. The two cell types were separated based on their marked differences in light‐scattering properties. Phagocytosing C2C12 cells were gated based on their Deep Red dye positivity and further analyzed for PIEZO1 expression. [file FSB2-40-e71578-s001.pdf]
